# Circulating Tumor DNA-Guided De-Escalation Targeted Therapy for Advanced Non−Small Cell Lung Cancer: A Nonrandomized Controlled Trial

**DOI:** 10.1001/jamaoncol.2024.1779

**Published:** 2024-06-13

**Authors:** Song Dong, Zhen Wang, Jia-Tao Zhang, Bingfa Yan, Chao Zhang, Xuan Gao, Hao Sun, Yang-Si Li, Hong-Hong Yan, Hai-Yan Tu, Si-Yang Maggie Liu, Yuhua Gong, Wei Gao, Jie Huang, Ri-Qiang Liao, Jun-Tao Lin, E-E. Ke, Zelong Xu, Xue Zhang, Xuefeng Xia, An-Na Li, Si-Yang Liu, Yi Pan, Jin-Ji Yang, Wen-Zhao Zhong, Xin Yi, Qing Zhou, Xue-Ning Yang, Yi-Long Wu

**Affiliations:** 1 Guangdong Lung Cancer Institute, Guangdong Provincial People’s Hospital, Guangdong Academy of Medical Sciences, Southern Medical University, Guangzhou, Guangdong, China; 2 Geneplus-Beijing Institute, Beijing, China; 3 Department of Hematology, First Affiliated Hospital, Jinan University, Guangzhou, Guangdong, China; 4 Chinese Thoracic Oncology Group, Guangzhou, Guangdong, China

## Abstract

**Importance:**

Uninterrupted targeted therapy until disease progression or intolerable toxic effects is currently the routine therapy for advanced non−small cell lung cancer (NSCLC) involving driver gene variations. However, drug resistance is inevitable.

**Objective:**

To assess the clinical feasibility of adaptive de-escalation tyrosine kinase inhibitor (TKI) treatment guided by circulating tumor DNA (ctDNA) for achieving complete remission after local consolidative therapy (LCT) in patients with advanced NSCLC.

**Design, Setting, and Participants:**

This prospective nonrandomized controlled trial was conducted at a single center from June 3, 2020, to July 19, 2022, and included 60 patients with advanced NSCLC with driver variations without radiologically detectable disease after TKI and LCT. The median (range) follow-up time was 19.2 (3.8-29.7) months. Data analysis was conducted from December 15, 2022, to May 10, 2023.

**Intervention:**

Cessation of TKI treatment and follow-up every 3 months. Treatment was restarted in patients with progressive disease (defined by the Response Evaluation Criteria in Solid Tumors 1.1 criteria), detectable ctDNA, or elevated carcinoembryonic antigen (CEA) levels, whichever manifested first, and treatment ceased if all indicators were negative during follow-up surveillance.

**Main Outcomes and Measures:**

Progression-free survival (PFS). Secondary end points were objective response rate, time to next treatment, and overall survival.

**Results:**

Among the total study sample of 60 participants (median [range] age, 55 [21-75] years; 33 [55%] were female), the median PFS was 18.4 (95% CI, 12.6-24.2) months and the median (range) total treatment break duration was 9.1 (1.5-28.1) months. Fourteen patients (group A) remained in TKI cessation with a median (range) treatment break duration of 20.3 (6.8-28.1) months; 31 patients (group B) received retreatment owing to detectable ctDNA and/or CEA and had a median PFS of 20.2 (95% CI, 12.9-27.4) months with a median (range) total treatment break duration of 8.8 (1.5-20.6) months; and 15 patients (group C) who underwent retreatment with TKIs due to progressive disease had a median PFS of 5.5 (95% CI, 1.5-7.2) months. For all participants, the TKI retreatment response rate was 96%, the median time to next treatment was 29.3 (95% CI, 25.3-35.2) months, and the data for overall survival were immature.

**Conclusions and Relevance:**

The findings of this nonrandomized controlled trial suggest that this adaptive de-escalation TKI strategy for patients with NSCLC is feasible in those with no lesions after LCT and a negative ctDNA test result. This might provide a de-escalation treatment strategy guided by ctDNA for the subset of patients with advanced NSCLC.

**Trial Registration:**

ClinicalTrials.gov Identifier: NCT03046316

## Key Points

**Question:**

Is a circulating tumor DNA (ctDNA)-guided adaptive de-escalation tyrosine kinase inhibitor (TKI) strategy clinically feasible for radiologically undetectable advanced non−small cell lung cancer (NSCLC) after targeted and local consolidative therapy?

**Findings:**

This nonrandomized controlled trial of 60 patients with advanced NSCLC found a median progression-free survival of 18.4 months, with 23% of patients requiring no additional TKI treatment; 52% receiving intermittent TKI retreatment guided by ctDNA or carcinoembryonic antigen before disease progression; and 25% receiving TKI retreatment due to disease progression.

**Meaning:**

These findings indicate that a ctDNA-guided adaptive de-escalation TKI treatment strategy is feasible for patients with advanced NSCLC and undetectable ctDNA for achieving complete remission after local consolidative therapy.

## Teaser

This nonrandomized controlled trial assesses the feasibility of using a circulating tumor DNA−guided adaptive de-escalation tyrosine kinase inhibitor strategy in patients with advanced non−small cell lung cancer to achieve complete remission after local consolidative therapy.

## Introduction

Targeted therapy has dramatically improved the clinical treatment efficacy and prognosis of patients with driver gene variation−positive non−small cell lung cancer (NSCLC), justifying its use as a standard first-line therapy.^1,2^ Nonetheless, nearly all patients inevitably develop resistance to targeted therapy. A common therapeutic strategy involves identifying mechanisms of resistance and initiating another therapy that targets the resistance mechanism (eg, *EGFR* T790M).^3,4,5^ Several randomized clinical trials have shown that local therapy may benefit patients with oligometastatic NSCLC, regardless of their treatment regimen and mutation status.^6,7,8^ Furthermore, local therapy may help patients achieve a disease-free status according to the Response Evaluation Criteria in Solid Tumors 1.1 (RECIST) criteria,^9^ similar to a complete response (CR). This raises the question of whether it is reasonable to suspend treatment in patients without radiologically detectable disease.

Treatment discontinuation is desirable for patients with a complete response or long-lasting stable disease who require long-term continuous treatment.^10,11^ Treatment discontinuation provides a treatment break or so-called drug holiday, which diminishes cumulative drug-related toxic effects and financial burden, thereby improving patient quality of life and potentially delaying drug resistance.^12,13,14^ The feasibility of this strategy has been confirmed in patients with chronic myeloid leukemia treated with tyrosine kinase inhibitor (TKI) therapy.^15,16^ For patients with solid tumors, 2 randomized phase 3 trials^11,12^ have indicated that despite no clinically meaningful difference regarding overall survival, discontinuation of TKI treatment may result in rapid disease progression. Thus, there is a need to explore new methods of treatment discontinuation to compare the survival benefits with the negative repercussions of continuous treatments. Some studies have proposed the use of an adaptive or intermittent treatment strategy.^17,18,19^

Given that tumors are typically monitored via radiographic imaging, reliable predictive factors are needed to identify patients who are most likely to benefit from treatment breaks and to assess treatment discontinuation and resumption schedules in patients without detectable disease. In a pilot clinical trial, prostate-specific antigen-guided adaptive therapy for metastatic castration-resistant prostate cancer showed promising efficacy.^17^ Tumor biomarker-guided adaptive therapy for advanced thyroid cancer is currently being tested in a phase 2 clinical trial (NCT03630120). Emerging data have shown that circulating tumor DNA (ctDNA) can be used as a potential molecular biomarker to predict residual disease in solid tumors and the risk of relapse is low for early-stage tumors treated with curative-intent therapy if ctDNA is undetectable.^20,21,22,23^ ctDNA has also been shown to precede radiologically detected progressive disease by 3 to 6 months.^20,21,24^ ctDNA analysis is usually performed to evaluate therapy and detect potential resistant genes in metastatic lung cancer.^25,26,27^ We hypothesized that for a highly selective subset of patients with advanced NSCLC without lesions, plasma ctDNA may be useful for detecting potential minimal residual disease (MRD). In this pilot study, we aimed to evaluate whether plasma ctDNA may serve as a useful biomarker for guiding adaptive de-escalation targeted TKI treatment in patients with NSCLC with driver variations.

## Methods

This was an exploratory proof of concept study based on a prospective trial^28^ that aimed to explore the efficacy of local consolidative therapy (LCT) for oligometastatic diseases after TKI treatment. This nonrandomized controlled trial was approved by the Research Ethics Committee of Guangdong Provincial People’s Hospital & Guangdong Academy of Medical Sciences. All patients provided written informed consent. The study followed the Transparent Reporting of Evaluations With Nonrandomized Designs (TREND) reporting guidelines.

### Study Design and Participants

Participant inclusion criteria were as follows: 18 years or older; histologically confirmed adenocarcinoma; inoperable stage IIIA, IIIB, or IV tumor (per the International Association for the Study of Lung Cancer *Staging Manual in Thoracic Oncology, eighth edition*^29^); *EGFR*-sensitive variant or anaplastic lymphoma kinase, c-ros oncogene 1, c-mesenchymal-to-epithelial transition alternation; Eastern Cooperative Oncology Group performance status 0 to 1; previous front-line targeted therapy; residual oligolesions eradicated by local therapy (eg, surgery or radiotherapy); and TKI therapy was paused postoperatively pending the results of ctDNA testing (within 2 weeks). The criteria for treatment discontinuation were as follows: no lesions (including target and nontarget lesions) after TKI and LCT according to the RECIST,^9^ undetectable ctDNA after LCT, and normal serum carcinoembryonic antigen (CEA) level after LCT. The complete study protocol is provided in Supplement 1.

**Figure 1.**
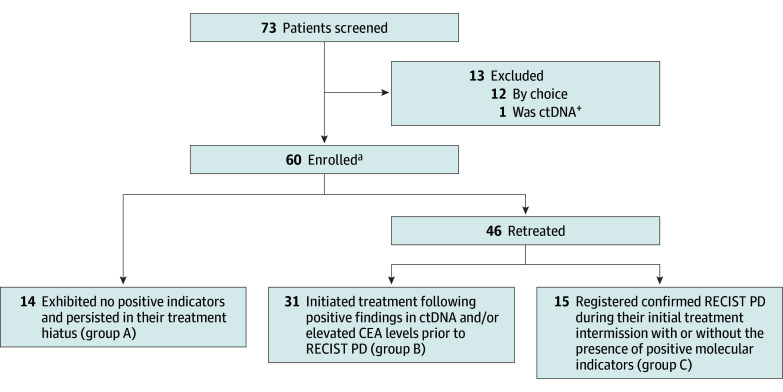
Summary of Participant Enrollment and Study Groups Group A included patients who exhibited no positive molecular indicators and persisted in their treatment hiatus; group B, patients who initiated treatment following the indication of positive ctDNA and/or elevated CEA levels before RECIST PD; and group C, patients who, during their initial treatment intermission, registered confirmed RECIST PD, with or without the presence of positive molecular indicators. CEA indicates carcinoembryonic antigen; ctDNA, circulating tumor DNA; PD, progressive disease; and RESIST, Response Evaluation Criteria in Solid Tumors, version 1.1.^9^ ^a^All enrolled patients had a negative test result for both CEA and ctDNA before the treatment break, and no radiologically measurable lesions.

From June 3, 2020, to July 19, 2022, we screened 73 patients with advanced-stage NSCLC with no lesions after undergoing previous TKI and LCT treatment. Of these, 60 patients were enrolled (Figure 1).

### Study Procedures

At treatment interruption, patients underwent the first surveillance of 6 weeks and follow-up every 3 months. Routine monitoring, including chest computed tomography (CT), brain magnetic resonance imaging, CEA, and plasma ctDNA. Abdominal CT or positron emission tomography−CT was performed at the physician’s discretion. Response to treatment was assessed according to the RECIST criteria.^9^ Patients required retreatment with prior TKIs if they experienced progressive disease or were positive for ctDNA or CEA, whichever occurred first. After 3 months of retreatment, radiologic examination, ctDNA, and CEA analyses were performed. If complete response was achieved and molecular indicators were negative, treatment was discontinued again. Methods of surveillance and treatment are described in the eFigure in Supplement 3.

Tumor tissue DNA was analyzed via next-generation sequencing of an oncoMRD-L panel of 1021 genes (GenePlus [China]) and plasma ctDNA was analyzed via next-generation sequencing of an oncoMRD-B panel of 338 genes (GenePlus), as previously described.^22^ Serum CEA levels were measured by our diagnostic laboratory, with CEA levels of less than 5.5 ng/mL (for µg/L, multiply by 1) considered to within the normal range.

### End Points

The primary end point assessed was progression-free survival (PFS), defined as the time from treatment discontinuation to initial identification of RECIST-based disease progression or death from any cause. The secondary end points were objective response, defined as the percentage of patients with a confirmed complete or partial response according to RECIST criteria^9^; time to next treatment (TTNT), defined as the duration from the initiation of the first treatment break to the alteration of the next treatment; and overall survival, defined as the time from the initiation of the first treatment break to death.

### Sample Size Calculations

The sample size was calculated using PASS software, version 15.0 (NCSS Statistical Software). As various TKI agents were used as first-line therapy in clinical practice and the historical control of PFS was set at 12 months,^1,5,30^ our study was designed with the expectation that we would observe a median (range) PFS improvement of 8 (12-20) months. We aimed to achieve a better median PFS than that of third-generation TKI used as first-line treatment.^5^ Assuming an enrollment time of 12 months and follow-up of 24 months, the sample size needed was determined to be 54 patients via the 1-sample log-rank test with a 1-sided significance level of .025 and 85% power. Considering a dropout rate of 10%, a total of 60 patients were required.

### Statistical Analysis

According to the study design, the following 3 outcomes were expected based on first retreatment triggers of enrolled patients at the cut-off time: group A, patients who had no positive indicators and continued treatment break; group B, patients who initiated retreatment after displaying positive ctDNA and/or elevated CEA levels before RECIST-based progressive disease; and group C, patients with confirmed RECIST-based progressive disease with or without positive molecular indicators during the first treatment break. The complete statistical analysis plan is available in Supplement 2. Kaplan-Meier analysis was performed to assess PFS, TTNT, and OS curves, with estimations of median and 95% CI values. Data analyses were performed from December 15, 2022, to May 10, 2023, using IBM SPSS Statistics version 25.0 (IBM Corp).

## Results

**Figure 2.**
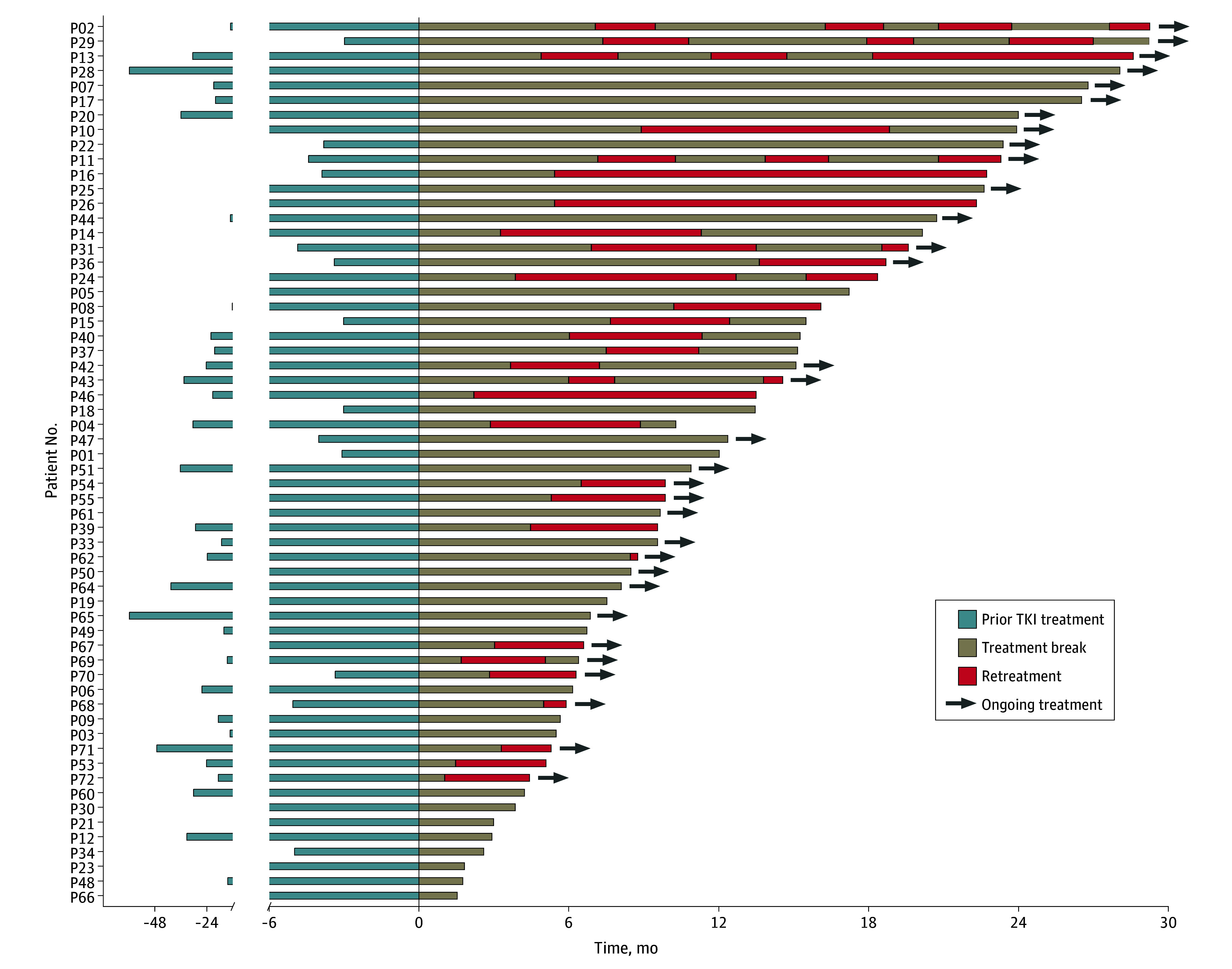
Timing and Duration of Tyrosine Kinase Inhibitor (TKI) Treatment, Breaks, and Retreatment Blue bars represent prior TKI treatment durations, beige bars represent treatment breaks, and red bars represent retreatment durations. Arrows indicate that the treatment type is ongoing.

The total study population included 60 patients (median [range] age, 55 [21-75] years; 33 females [55%] and 27 [45%] males) who had been diagnosed with lung adenocarcinoma with driver gene-sensitive variations. Baseline patient characteristics and treatment information are summarized in the Table and additional details are provided in the eTable in Supplement 4. All 60 participants had received TKI before enrollment: 50, first-line TKI treatment and 10, second-line TKIs. The median (range) duration of the preceding TKI therapy was 12.0 (3.0-65.9) months. By the cut-off date of November 30, 2022, all patients had received at least 1 treatment break. The median (range) follow-up time after treatment cessation was 19.2 (3.8-29.7) months (Figure 2).

**Table.** Baseline Demographic and Clinical Characteristics of Study Participants

Characteristic	No. (%)
Total participants	60 (100)
Female	33 (55)
Male	27 (45)
Age, median (range), y	55 (21-75)
Tumor stage^a^	
III	8 (13)
IV	52 (87)
Smoking	
Yes	11 (18)
No	49 (82)
Pathology results	
Adenocarcinoma	60 (100)
Actionable gene variation	
*EGFR* 19del	29 (48)
*EGFR* 19 plus T790M	6 (10)
*EGFR* 21 L858R	18 (30)
*EGFR* 21 plus T790M	3 (5)
*ALK* fusion	3 (5)
*ROS1* fusion	1 (2)
Target-drug pair	
EGFR-TKI I	17 (28)
EGFR-TKI II	16 (26)
EGFR-TKI III	13 (22)
EGFR-TKI I plus III	7 (12)
EGFR-TKI II plus III	3 (5)
ALK-TKI I	1 (2)
ALK-TKI II	3 (5)
Treatment lines	
First	50 (83)
Second	10 (17)
Organ(s) with metastasis	
1	45 (75)
2	9 (15)
≥3	6 (10)
Location of metastasis, No.	
Lung	26
Bone	18
Pleura	17
Brain	8
Lymph	8
Pericardium	2
Ovary	1
Diaphragm	1
Local consolidative therapy	
Radiotherapy	2 (3)
Surgery	54 (90)
Surgery and radiotherapy	4 (7)

Abbreviations: *EGFR*, epidermal growth factor receptor; TKI, tyrosine kinase inhibitor; *ALK*, anaplastic lymphoma kinase; *ROS1*, c-ros oncogene 1.

^a^ Per the International Association for the Study of Lung Cancer *Staging Manual in Thoracic Oncology, eighth edition*.^29^

### Progression-Free Survival

**Figure 3.**
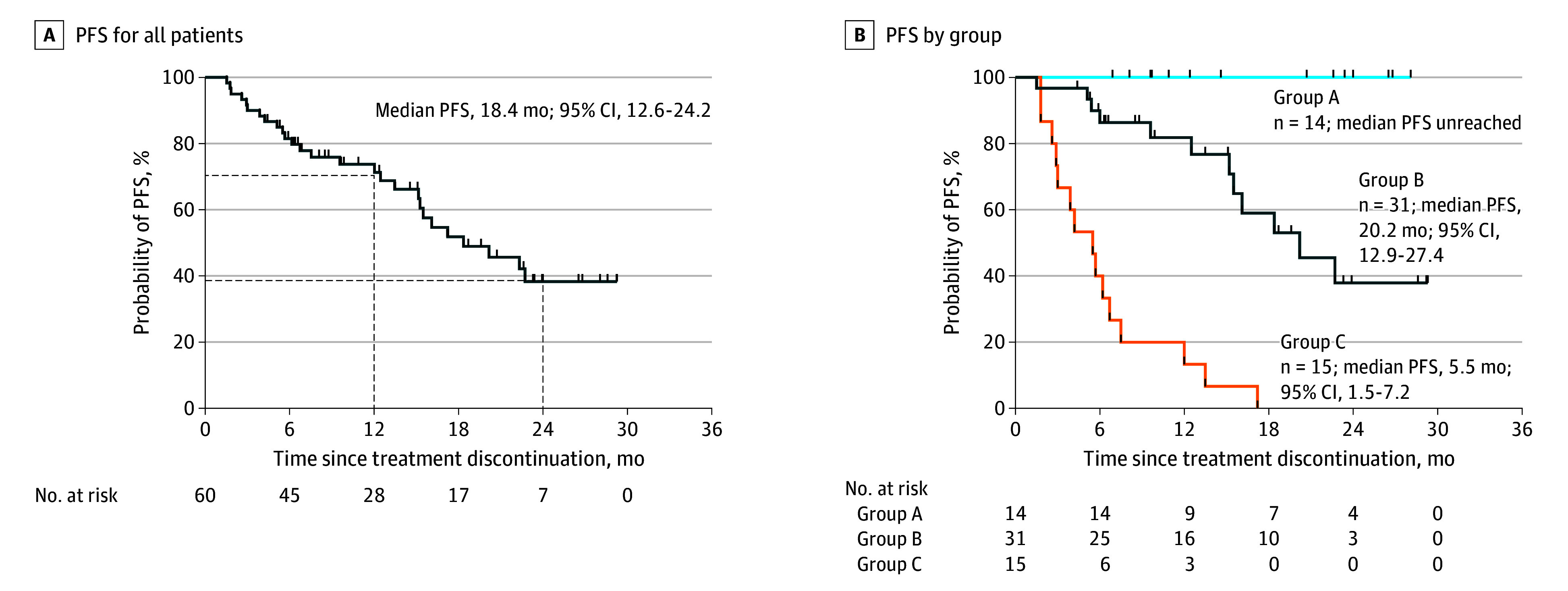
Progression-Free Survival (PFS) for All Participants and by Study Group A, PFS of all patients. B, PFS for patients classified with the following indicators of retreatment: group A, no positive molecular indicators; group B, positive molecular indicators; and group C, radiographic progression.

At the end of the study period, 27 patients (47%) had experienced disease progression, with a median PFS (mPFS) of 18.4 (95% CI, 12.6-24.2) months (Figure 3A). Twelve- and 24-month FPS rates were 67.7% (95% CI, 53.5%-78.5%) and 40.2% (95% CI, 24.3%-55.6%), respectively. The median (range) total treatment break duration was 9.1 (1.5-28.1) months, and no patient died during treatment discontinuation. According to the study protocol, 3 outcomes (groups A, B, and C) were possible based on triggers of the first retreatment. Group A was composed of the 14 participants (23%) who remained in treatment break with a median (range) treatment break of 20.3 (6.8-28.1) months. Group B was composed of the 31 participants (52%) who initiated retreatment after displaying positive molecular indicators before RECIST progressive disease, including detectable ctDNA (26 patients) and elevated CEA level (5 patients). Of these 31 patients, 12 (39%) experienced progression in subsequent retreatment (n = 3) or break interval (n = 9) with a median PFS of 20.2 (95% CI, 12.9-27.4) months. They had a median (range) of 2 (2-4) treatment breaks and the median (range) total treatment breaks duration was 8.8 (1.5-20.6) months. Group C was composed of the 15 participants (25%) who experienced confirmed progressive disease (according to RECIST criteria) and who underwent retreatment with a median PFS of 5.5 (95% CI, 1.5-7.2) months (Figure 3B). Among these 15 patients, ctDNA was undetectable in 9, including 6 patients who exclusively exhibited brain metastases.

### Time to Next Treatment and Overall Survival

**Figure 4.**
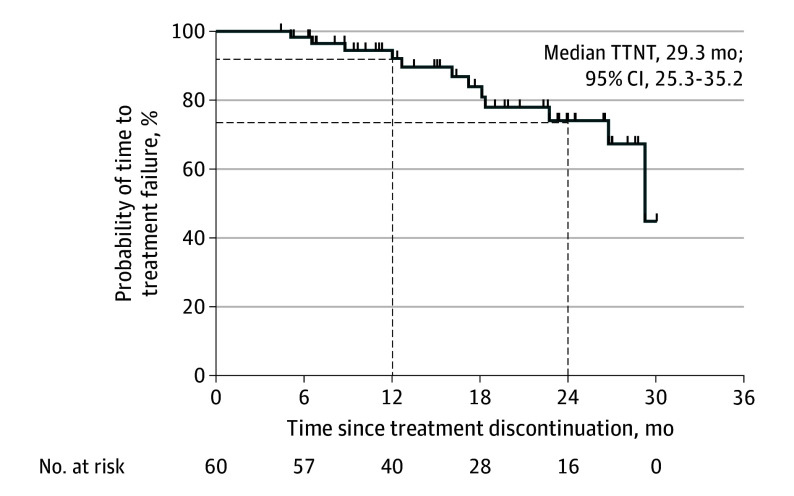
Time to Next Treatment (TTNT) Failure and Duration of Discontinuation

According to the trial protocol (Supplement 1), TTNT was used to evaluate the actual TKI treatment duration. In total, 12 patients eventually experienced disease progression while receiving TKI retreatment and were administered next-line treatment by their physicians. The median TTNT from the initiation of the first treatment break was 29.3 (95% CI, 25.3-35.2) months, with 12- and 24-month TTNT of 92.2% (95% CI, 80.2%-97.0%) and 74.1% (95% CI, 56.2%-85.5%), respectively (Figure 4). The median overall survival data were immature.

### Efficacy of Retreatment and Toxic Effects

For the 27 patients who experienced progressive disease, an important issue was the response to retreatment with prior TKIs. Besides 3 patients in group B who initiated retreatment due to positive molecular indicators and confirmed radiographic progression in subsequent retreatment, the objective response to retreatment with prior TKIs was evaluated in 24 patients (9 in group B and 15 in group C) who experienced a first disease progression during treatment break intervals. Among these 24 patients, 12 achieved CR, 11 achieved PR, and 1 had stable disease. The objective response rate was 96% (95% CI, 87.7%-100%). Twelve of 24 patients who achieved sufficient tumor regression opted to discontinue TKI treatment. In group B, ctDNA was undetectable in 96% of patients (25 of 26) after 3-month retreatment with prior TKI treatments, and CEA reached a normal level in 3 of 5 patients. Two other patients exhibited decreased CEA levels that persisted above the normal range. For patients who received TKI retreatment, grade 1 to 2 adverse events included rash (n = 7), paronychia (n = 2), and arrhythmia (n = 1); no grade 3 or worse adverse event was observed.

### Metastasis Pattern and Subsequent Treatment Options

Among 27 patients who experienced progressive disease, 9 (33%) developed intrathoracic metastases, 11 (41%) developed extrathoracic metastases, and 7 (26%) developed both. Additionally, 3 patients had growing oligometastatic nodules in the lungs and underwent a second wedge resection per the decision of a multidisciplinary team, and 1 other patient received rib radiotherapy locally. Of these 27 patients, 12 (44%) eventually experienced progression while receiving retreatment with prior TKI and were instructed by their physicians to initiate next-line treatment; among them, 7 received third-generation *EGFR* TKI; 4 received chemotherapy; and 1 received a combination therapy of erlotinib and bevacizumab.

## Discussion

To our knowledge, this is the first study to provide evidence showing that an adaptive de-escalation TKI treatment strategy guided by ctDNA is feasible for certain subsets of patients with NSCLC harboring driver gene variations. The findings of our study suggest that at least 75% of patients with driver gene alternation could benefit from this drug holiday strategy. Moreover, 23% did not relapse after a median follow-up duration of 19.2 months. For the overall population, the mPFS of 18.4 months exceeded the first-line third-generation *EGFR*-TKI reference mPFS (19-22 months).^5,31,32,33,34^

Multiple studies have provided evidence that patients with NSCLC who have a limited number of metastases may achieve long-term survival after aggressive local therapy.^6,7,8^ Two retrospective studies^35,36^ have reported the PFS of patients who received first-line continuous TKI with LCT for NSCLC with driver gene variants and oligometastatic disease. A PFS of 20.6 months was reported for patients who received local ablative therapy at both primary tumor and oligometastatic sites,^35^ and another study revealed that 12 patients who received *EGFR*-TKI and LCT had a PFS of 36 months.^36^ In contrast to previous standard continuous TKI treatment, we proposed a ctDNA-guided adaptive de-escalation treatment strategy that demonstrated a PFS of 18.4 (95% CI, 12.6-24.2) months. Considering the duration of TKI therapy (12 months) before enrollment and long-lasting responses after retreatment, we speculate that our adaptive de-escalation TKI treatment strategy may be associated with similar or more favorable outcomes compared with these studies.^35,36^

This study found that 23% of the patients (group A) showed no positive indicators, yet they could achieve a treatment break of 20.3 (6.8-28.1) months. These patients received no cancer therapy and close surveillance. This is an important observation because continuous TKI therapy is the standard approach for treating these patients in clinical practice. We believe that this group may represent a subset of patients with an indolent phenotype who may benefit greatly from an adaptive treatment strategy.

Previous studies have shown that combination of ctDNA analysis and radiographic imaging may improve the early detection of potential progression of NSCLC compared with imaging alone.^37^ In our study, more than half (26 of 46 patients) of the retreatment was guided by the detection of ctDNA. Next-generation sequencing analysis was performed 3 months after retreatment, and ctDNA was undetectable in almost all plasma samples of these patients (96%; 25 of 26 patients). Therefore, we speculate that this retreatment is effective. Patients in group B had a PFS of 20.2 months. The PFS of patients treated with TKIs guided by ctDNA-based molecular biomarkers is encouraging. Additionally, the adaptive de-escalation strategy provided patients with a treatment break. The median cumulative treatment-free interval was 8.8 months for patients of group B. Although no formal comparison was conducted to evaluate treatment toxic effects or quality of life in this single-group study, it is conceivable that an adaptive de-escalation treatment strategy may potentially reduce economic burden, diminish toxic effects, and improve quality of life.

In this study, patients in group C experienced progressive disease with a PFS of 5.5 months. The determination of clinicopathological features and biomarkers of a high-risk subgroup is warranted. When treatment is discontinued, retreatment efficacy is an important concern. Previous studies have shown that treatment interruption does not affect the response rate of retreatment.^11,18,19,38^ In our study, the response rate was 96%, suggesting that treatment interruption did not compromise the efficacy of retreatment with prior TKIs. These results indicate that patients will not be at increased risk of adverse outcomes after undergoing temporary treatment disruptions.

The utility of treatment discontinuation depends on the ability of physicians to identify microscopic remnants and hidden metastases. Emerging evidence has indicated the efficacy of blood-based ctDNA analysis for detecting MRD.^20,39,40^ The sensitivity of MRD depends greatly on the amount of ctDNA released into the bloodstream.^41^ In a previous study, we showed that ctDNA analysis is ideal for predicting the absence of disease during postoperative monitoring.^22^ In this study, 26 patients (57%) received retreatment due to the detection of ctDNA, highlighting the importance of ctDNA analysis in identifying MRD in advanced NSCLC after TKI discontinuation. Among the 9 patients who experienced disease progression without detected ctDNA, 6 patients with brain metastasis only. This suggested the limit of detection of current technologies, particularly for some metastatic sites, such as the cerebrum.^22^ As ctDNA analysis platforms continue to develop, future technical advances will improve the sensitivity of ctDNA detection. An adaptive de-escalation therapeutic strategy based on high-sensitivity ctDNA analysis deserves to be verified across a broad spectrum of malignancies and anticancer agents.

CEA was not recommended as a biomarker to identify patients at increased risk of relapse because CEA lacks sensitivity and specificity.^42,43^ However, if CEA levels are elevated at baseline in patients with NSCLC, it is worth noting that a significant association has been demonstrated between increases in CEA levels and radiological progression.^24,44^ A strategy of incorporating ctDNA and protein biomarkers, including CEA, has been used to screen for different cancers.^45^ In our study, 26 patients had elevated baseline CEA levels that decreased to normal levels before enrollment; 13 showed elevated CEA levels during initiation of the first retreatment. Furthermore, 5 patients had elevated CEA levels only as a trigger to the retreatment. In these 5 patients, CEA levels decreased to normal levels in 3 patients after retreatment, and 2 patients exhibited a decrease in CEA levels that persisted higher than the normal value before relapsing 14 and 18 months after retreatment with prior TKIs. This finding suggests that CEA is a potentially useful indicator when measured in combination with ctDNA during treatment breaks.

### Limitations

This study had some limitations. First, baseline ctDNA values before targeted therapy (pretreatment) were not available; therefore, nonshedders may have potentially affected results. Second, this was a single-intervention study at a single center; lacking a control group could lead to potential selection bias. Therefore, these study results must be confirmed by a randomized clinical trial to truly determine the efficacy of the treatment strategy. Third, the follow-up duration was limited because many patients remained in the retreatment or treatment break interval. A longer follow-up duration would provide further information on overall survival and PFS outcomes after treatment.

## Conclusions

The findings of this nonrandomized controlled trial provide valuable information regarding the potential utility of planned adaptive de-escalation therapy in the treatment of patients with advanced NSCLC. The use of reliable ctDNA-based assays enables the accurate monitoring of patient status after systemic therapy and LCT.

## Notes

Supplement 1. Trial Protocol

Supplement 2. Statistical Analysis Plan

Supplement 3. eFigure. Schematic flow chart of the study

Supplement 4. eTable. Clinical characteristics and triggers of first retreatment for each patient

Supplement 5. Data Sharing Statement
