# Supplementary material for: Circulating Tumor DNA-Guided De-Escalation Targeted Therapy for Advanced Non−Small Cell Lung Cancer: A Nonrandomized Controlled Trial
Source: JAMA Oncol. 2024 Jun 13;10(7):932–40. doi: 10.1001/jamaoncol.2024.1779 (PMC12312504; doi:10.1001/jamaoncol.2024.1779)
Supplement: Supplement 1. — Trial Protocol [file jamaoncol-e241779-s001.pdf]

Study Protocol

Local Consolidative Therapy in Patients with Advanced Stage Non-small Cell Lung Cancer that Does Not Progress after Front Line Systemic Therapy

Trial Number:	CTONG1602
Version:	Version: 3.3
Registration Number:	NCT03046316
IRB Approval Number:	IRB Approval Number: No. GDREC2016416H
Sponsor:	Guangdong Association of Clinical Trials/Chinese Thoracic Oncology Group (GACT/CTONG)
Principal investigator:	Zhen Wang

Table of content

1 Study introduction....4
1.1 Study background 4
1.2 Study rationale 6
1.3 Exploratory Study 6
2 Study objectives and study endpoints....6
3 Study design 7
3.1 Description of Study Design 7
3.2 Definition of Study End 7
3.3 Counting the Number of Metastatic Lesions 7
3.4 Definition of Local Therapy 8
3.5 Duration and Sample Size 8
3.6 Ethical Application 8
4 Study Population....8
4.1 Inclusion Criteria 8
4.2 Exclusion Criteria 9
5 Study Procedure and Evaluation 10
5.1 Study Treatment 10
5.2 Study Flow Chart 11
5.3 Test Schedule 12
5.4 Modalities of Efficacy Assessment 13
5.5 Content of patient outcome assessment 14
5.5.1 Primary endpoint 14
5.5.2 Secondary endpoints 14
5.5.3 Security assessment 14
5.5.4 Quality of life scores 14
6 Security Monitoring and Reporting 15
6.1 Adverse Event Definition 15
6.2 Adverse Events Assessments 15
6.3 Reporting of Adverse Events 15
6.4 Adverse Event Severity 16
6.5 Serious Adverse Event Definition 16
6.6 Reporting of Serious Adverse Events 16
6.7 No SAE Conditions 16
7 Data Collection and Management 16
7.1 Traceability of Data 16

7.2 Database Design and Creation 16
7.3 Data Verification 16
7.4 Data Quality Control 16
7.5 Control of Bias and Confounding Variables 17
7.5.1 Control Methods for Bias and Confounding Variables 17
7.5.2 Control Methods for Information Bias 18
8 Ethical Considerations and Management Procedures 18
8.1 Regulatory and Ethical Compliance 18
8.2 Responsibilities of the Investigator and IRB/IEC/REB 18
8.3 Informed Consent Procedures 18
8.4 Publication of Study Protocol and Results 19
8.5 Articles Published by the Investigator 19
8.6 Storage of Study Documents and Records and Retention of Documents 19
8.7 Confidentiality of Study Documents and Patient Records Storage of Study 20
9 Protocol Amendments 20
References 21

1 Study background and rationale

1.1 Study background

Non-small cell lung cancer is the leading cause of cancer deaths; about half of the patients have metastases at the presentation time [1]. In some of these patients, local therapy achieved prolonged survival in some subset of NSCLC patients. Theoretically, local therapy for all metastatic sites may prolong PFS (Progression-Free Survival) and OS (Overall Survival) and may even be potentially curative in a small number of patients [2].

It is well known that the tumor's biological characteristics genuinely affect the prognosis of metastatic lung cancer, and the prognostic impact of local therapy is unknown. However, for now, local therapy's complications and mortality rates are so low that they can be applied selectively. Evidence supporting local therapy is mainly limited to retrospective studies and a few single-arm prospective studies reporting safety and efficacy. Without robust supportive data, local therapy of metastatic lung cancer can only be guided by clinical judgment and physician experience. usually, surgery and SBRT are the primary method for single metastasis disease under the multidisciplinary team discussion [2].

For NSCLC patients with single brain metastasis, adrenal gland metastasis, or contralateral lung metastasis. local therapy for the metastatic disease is recommended. Surgical resection may also be considered in certain patients with solitary metastases in other organs, but the efficacy of local therapy for prolongs survival is uncertain. the clinical benefit of surgery in such patients remains controversial [3]. Shimada, Y. et al. conducted a retrospective analysis of 272 patients with recurrent distant metastases after surgical resection of lung cancer from 2000 to 2011. Overall disease-free survival was 14.1 months. The 2-year postoperative recurrence rate was 73.5%. Two groups were categorized according to the type of postoperative recurrence: oligo-metastases and multi-metastasis. Oligometastases are defined as 1 or 2 organs with 1 to 5 distant metastatic lesions. Of these, 47 patients with oligo-metastases received local therapy, including surgery, radiotherapy, and systemic sequential therapy, and chemoradiotherapy. In the multivariate analysis, patients with oligometastatic disease, adenocarcinoma and long DFS showed a better prognosis. Overall, the presence of oligometastases after surgery has a good prognosis and personalized therapy for patients with oligometastases is essential [4].

No prospective study is reported at present that directly comparing surgery and radiotherapy for advanced lung cancer. SABR is well accepted for intracranial lesions and extracranial metastases and is increasingly used in treatment of metastatic lung cancer [5]. Phase I/II clinical trials have revealed disease control rates of 70% to 90% in lung, liver, spine, and multiple metastatic lesions with the use of massively fractionated radiotherapy, with a less than 10% incidence of toxicity of grade 3 or higher [6, 7]. The clinical trials on surgery for single brain

metastases stage IV NSCLC confirmed the feasibility of the local therapy[8, 9]. About a quarter of stage IV NSCLC has brain metastases. In about 10% of metastatic lung adenocarcinomas, the brain is the only site of metastasis. patients are encouraged to undergo radical treatment for both the primary lesion and metastatic lesions. Multiple brain metastases are not an absolute contraindication to surgery, but three or fewer lesions are recommended [10].

Brain metastases can be treated with surgical resection or stereotactic radiosurgery. The 5-year overall survival after radical treatment for single brain metastases and primary lesions is 15% [8, 9]. surgery for oligometastatic lesions, whether intracranial or extracranial, has confirmed the efficacy of prolonging survival, especially in patients with no lymph nodes metastases, adenocarcinomas, and small lung metastatic lesions. The combination of targeted therapy and radiotherapy has also shown promising results in patients with driver gene mutant oligometastases [11].

surgery for adrenal metastatic lesions in lung cancer patients provides a survival benefit [12]. Selective isolated adrenal metastases have a 25% survival rate with complete surgical resection [13]. Surgical mortality is extremely low, and most patients die from disease progression. Lung cancer patients with single pancreatic metastasis who received radical surgery has got clinical benefits [14].Interventional ablation has been used for local therapy of lung cancer, but it is usually been used in certain inoperable patients. The RAPTURE study demonstrated that lung cancer patients with high-risk factors who underwent ablation had 2-year and overall survival rates of 48% and 73%, and lung function was largely unaffected [15].

A prospective randomized controlled study of local therapy of advanced NSCLC oligometastases (≤ 3 metastatic lesions) was conducted previously. Patients with oligometastatic lung cancer without progression on systemic therapy were randomized 1:1 to the experimental group (local treatment ± maintenance therapy) or the control group (maintenance therapy or observation). From November 28, 2013, to January 19, 2016, 74 patients were enrolled. The trial was discontinued early after 49 patients completed randomization in the experimental group as the interim analysis showed a significant clinical benefit. Twenty-five cases had local therapy, and 24 cases had maintenance therapy. After a median follow-up of 12.4 months, the median PFS was 11.9 months (90% CI. 5.7-20.9) and 3.9 months (90% CI. 2.3-6.6) in the local therapy group and the maintenance group (HR:0.35, 90% CI. 018 – 0.66, p=0.0054), respectively, which has met the primary endpoint of the study [16]. At the updated follow-up of 38.8 months (28.3-61.4), the local treatment group maintained a PFS of 14.2 months (95% CI: 7.4-23.1), while the maintenance treatment group had a PFS of 4.4 months (95% CI: 2.2-8.3) (p=0.022). There was a survival benefit in the local therapy group, with an OS of 41.2 months (95% CI. 18.9-NR) and 17.0 months in the maintenance treatment group (95% CI. 10.1-39.8) (p=0.017). The study met

the secondary research outcome OS. Overall survival after disease progression remained beneficial in the local therapy group compared to the observation group (37.6 vs. 9.4, p=0.034). Twenty patients in the maintenance group had a median OS of 17 months (95% CI:7.8-NR) [17].

1.2 Study rationale

The following hypotheses have been proposed for the OS benefit from combination of local therapy and systemic therapy. In those patients with stable disease after systemic therapy, residual drug-resistant cancer cells are difficult to eliminate and become a source of disease progression [18]. However, local therapy, such as local radiotherapy, can enhance the efficacy of systemic therapy. On the other hand, it has been observed that residual tumors can promote the growth of distant tumor micrometastases through immunosuppressive and pro-angiogenic effects in preclinical study [19]. Thus, local therapy may retard the development of distant metastases by reducing residual drug-resistant cancer cells.

This study aims to investigate the efficacy of local therapy in combination with systemic therapy in Chinese NSCLC patients. The primary objective of this study is to explore better therapeutic strategies for improving outcomes of patients with advanced lung cancer.

1.3 Exploratory Study

During the follow-up periods, tumors are typically monitored via radiographic imaging, but the strategy of local therapy in combination with systemic therapy would achieve a situation of undetectable disease for some advanced NSCLC patients, and this give rise to a question of how to evaluate the potential minimal tumor progression ahead of radiographic imaging. Emerging data have shown that ctDNA can be used as a potential molecular biomarker to predict residual disease in solid tumors. The risk of relapse is low for early stage tumors treated with curative-intent therapy if ctDNA is undetectable [20-22]. and ctDNA has been shown to precede radiological progressive disease (PD) by 3–6 months [20,21,23]. ctDNA analysis is usually performed to evaluate therapy and detect potential resistance genes in metastatic lung cancer [24-27]. plasma ctDNA may be useful for guiding personalized patient management. In this study, we initiate an exploratory part aims to examine whether plasma ctDNA may serve as a biomarker for guiding adaptive de-escalation targeted TKI treatment for NSCLC patients with no detectable disease after local therapy.

2 Study objectives and study endpoints

**Study objectives**	**Study endpoints**
Primary study objective<br>To investigate the efficacy of local	Primary study endpoint<br>PFS:

therapy in combination with systemic therapy in Chinese NSCLC patients.	
Secondary study objectives<br>To further evaluate the efficacy of local therapy in combination with systemic therapy, [redacted]	Secondary study endpoints<br>OS;<br>Safety;<br>Quality of life
Exploratory study objectives<br>to examine whether plasma ctDNA may serve as a biomarker for guiding adaptive de-escalation targeted TKI treatment for NSCLC patients with no detectable disease after local therapy	PFS:<br>TTNT

PFS, progression-free survival; OS: overall survival; TTNT, time to next treatment

3 Study design

3.1 description of study design

This study is a prospective real-world study designed to explore the efficacy and survival benefit, as well as the safety of local therapy in progression-free patients with advanced Non-Small-Cell Lung Cancer (Stage IV, 8th edition TNM staging) who received first-line systemic therapy (chemotherapy, targeted therapy, or immunotherapy), and received local therapy (surgery, radiotherapy, or interventional therapy). The exploratory Study subgroup: For driver gene positive patients who underwent surgery for all the primary and/or metastatic lesions with no detectable disease on radiographic imaging after surgery. ctDNA analysis will be performed for the patients. If the ctDNA, CEA and imaging remain negative, the patients will be enrolled into the de-escalation targeted therapy group; systemic targeted therapy will be discontinued, and if any one of these indicators becomes positive, then targeted therapy will be initiate.

3.2 Definition of end of study

The end of the study is defined as the completion of survival follow-up of all patients in all treatment cohorts or early termination of the study. The final study data analysis will be performed at the end of the study.

3.3 Counting the number of metastatic lesions

primary lesions as well as each metastatic lesion were counted separately. Pre-treatment lesions were no longer counted after they disappeared on CT scans following systemic therapy, and all mediastinal metastatic lymph nodes (N1-N3)

including supraclavicular lymph nodes were recorded as one lesion. Those who required immediate local therapy prior to systemic therapy (e.g., surgery or radical radiotherapy for brain metastases) and who received systemic therapy for non-PD maintenance for more than 3 months were allowed into this study, but the metastatic lesions were counted.

3.4 Definition of local therapy

Patients received local therapy with definitive efficacy (that can eradicate local lesions), including surgery, radiation therapy, and interventional therapy. Palliative care aimed at reducing symptoms was excluded. Local therapy could target one or more lesions and must include at least 1 major lesion.

3.5 Duration and sample size

[redacted] A total of 60 cases are planned for the de-escalation group, and the study initiated in June 2020. The planned enrollment will continue for 24 months.

3.6 Ethical application

the study protocol was submitted to and approved by the Hospital Ethics Committee. All patients are required to sign an informed consent form before enrollment.

4 Study population

4.1 Inclusion criteria

1. Age ⩾18 years, life expectancy ⩾12 weeks
2. Histologically or cytologically confirmed advanced stage (inoperable stage III, stage IV, International Association for the Study of Lung Cancer 8th edition) NSCLC
3. Driver genes mutation analysis is necessary (at least for the EGFR and ALK)
4. The status of non-PD (CR, PR, or SD) last for at least 3 months after first-line systemic therapy (chemotherapy, targeted therapy, or immunotherapy). No more than five lesions after systemic therapy.
5. The patient is considered to be suitable for local therapy by the researchers (the following conditions in parentheses are for the researcher's reference and may be met or partially met).
6. ECOG score ⩽ 2
7. Have adequate organ function, defined as follows: liver function:

8. In the absence of liver metastases, serum aspartate aminotransferase (AST) as well as serum alanine aminotransferase (ALT) ≤ 3 times upper limit of normal (ULN) or ≤ 3 times baseline if baseline is abnormal. In the case of hepatic metastases, AST and ALT ≤ 5-fold ULN. Total serum bilirubin ≤ 1.5 times ULN
9. Bone marrow function:
10. Peripheral blood leukocyte count ≥3.0X109/L, platelets ≥75X109/L, hemoglobin ≥8.0 g/dL. Renal function: serum creatinine ≤ 1.5 ULN or creatinine clearance (based on the modified Cockcroft-Gault formula) ≥ 60 ml/min.
11. with coagulation function within normal limits]
12. Women of childbearing potential who have had a negative pregnancy test (urine or serum) within 7 days prior to local therapy and who agree to use appropriate contraception.
13. Men whose wives are of childbearing age must agree to use suitable contraception during and for 12 weeks after treatment.
14. Signed written informed consent.
15. Able to comply with study requirements and follow-up procedures.

For the subgroup of de-escalation targeted therapy

16. surgery as the local therapy for all the primary and/or metastatic lesions.
17. ctDNA analysis shows negative after local therapy.
18. Normal CEA level.

4.2 Exclusion criteria

1. Patients with stage I-III NSCLC who have relapsed after prior radical treatment (surgery or concurrent radiochemotherapy) followed by systemic therapeutic antitumor therapy are not eligible for enrollment.
2. First-line systemic therapy without PD for more than 3 months, but PD occurred during maintenance therapy.
3. Achieving CR through systemic treatment.
4. Malignant pleural effusion or significant pleural cavity effusion that cannot be controlled after drainage and systemic treatment.
5. Patients with a history of secondary malignant tumors within five years (except for adequately treated cervical carcinoma in situ, basal cell carcinoma, or squamous cell skin carcinoma).
6. Those who had undergone palliative surgery for advanced lung cancer prior to systemic therapy were eligible for enrollment, but palliative therapy could not be credited as radical treatment for the lesion. Those who require immediate local therapy prior to systemic therapy (e.g., surgery or radical radiotherapy for brain metastases) and who receive systemic therapy for non-PD maintenance for more than 3 months may be entered into this study, but

the metastatic lesions will need to be counted.

7. Patients who had contraindications to local therapy (including surgery, radiotherapy, or intervention) assessed by the researchers.
8. Patients with a personal history of human immunodeficiency virus (HIV) that can be transmitted through blood or other bodily fluids.
9. Pregnant and lactating women.
10. Women of childbearing age and sexually active men who were unwilling to use contraception during the study period.
11. The patient has a mental illness or other condition that causes noncompliance with treatment.
12. Patients who refuse to sign an informed consent form.

For the subgroup of de-escalation targeted therapy

13. Refuse the ctDNA analysis or the ctDNA is positive after surgery.
14. Elevated CEA above the normal level.

5 Study procedure and evaluation

5.1 Study Treatment

Patients eligible for enrollment were discussed in MDT to determine local therapy options and signed informed consent to enter this study for local therapy. After completion of local therapy, [redacted] For the subgroup of de-escalation targeted therapy, the patients discontinue the targeted therapy.

5.2 Study flow chart

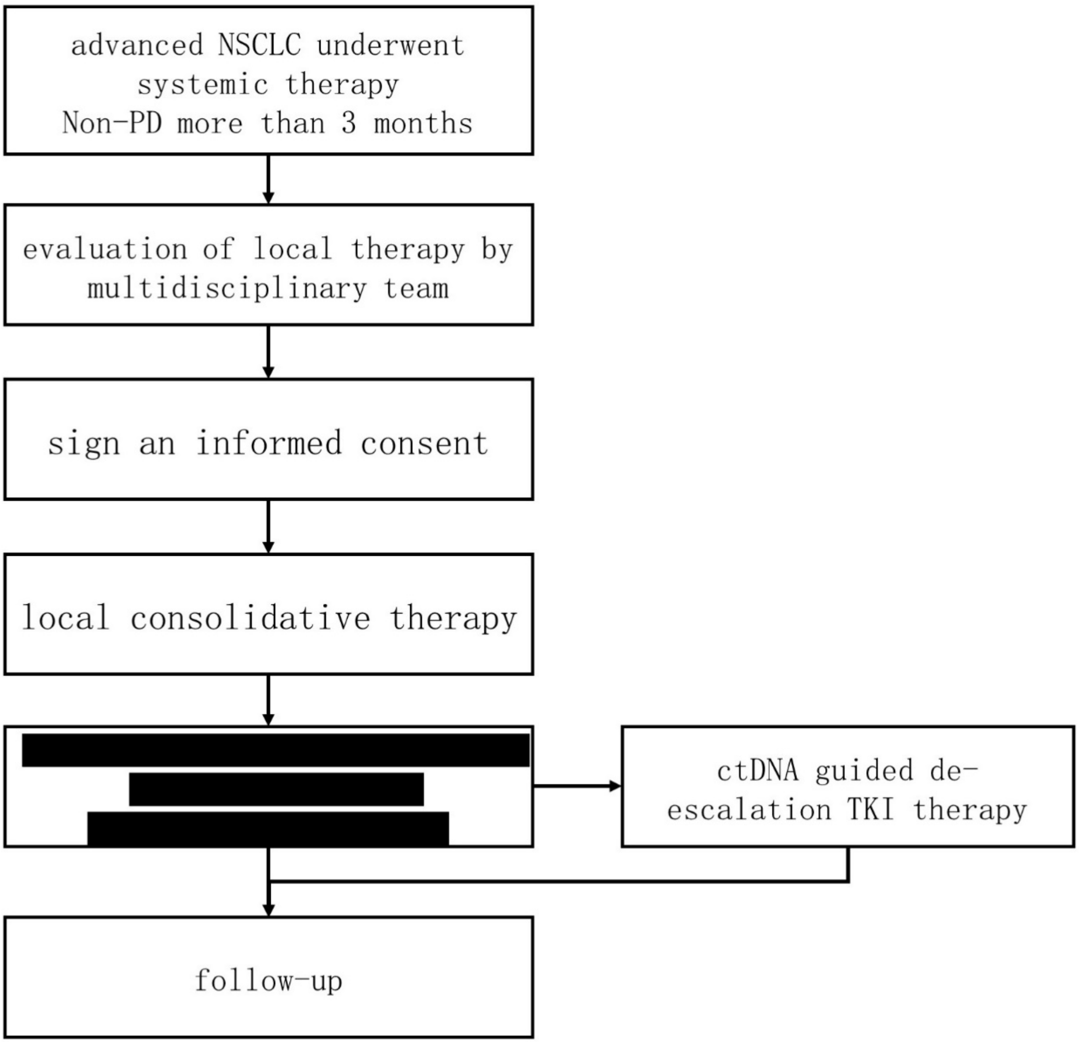
advanced NSCLC underwent
systemic therapy
Non-PD more than 3 months
evaluation of local therapy by
multidisciplinary team
sign an informed consent
local consolidative therapy
[redacted]
ctDNA guided de-
escalation TKI therapy
follow-up

5.3 Test schedule

Test schedule

Entry	Baseline/Befoe Local Therapy (Within 14 Days)	Treatments	After Local Treatment (4 Weeks ± 7 Days)	[redacted]-de-escalation(Every 12 Weeks ± 7 Days	Disease Progression (Window Period ± 7 Days)	Post-Progression Survival Follow-Up (Every 3 Months ± 15Days)
informed consent	X					
Demographic information	X					
medical history	X	X	X	X	X	
clinical examination	X	X	X	X	X	
weight	X	X	X	X	X	
(a person's) height	X					
(body) temperature	X	X	X	X	X	
pulse (both medical and figurative)	X	X	X	X	X	
blood pressure	X	X	X	X	X	
ECOG PS	X	X	X	X	X	
adverse event	X	X	X	X	X	
routine blood test	X	X	X	X	X	
blood biochemistry	X	X	X	X	X	
blood coagulation	X					

function						
urinalysis	X	X	X	X	X	
ctDNA			X	X	X	
Radiological assessment	X	X	X	X	X	
Questionnaire (Quality of Life Scores LCSS)	X			X	X	
condition				X	X	~~X~~

History: includes clinical diagnosis, symptoms, past clinical history.
Chemotherapy regimen: record details of the regimen including dose adjustments.
Targeted therapy: document the targeted therapy regimen as well as the dose.
Local therapy: local therapy within 1 month after signing the informed consent form. Detailed records of local therapy plan, collection of surgical records, postoperative pathology; radiotherapy records, including time, site, and dose of radiotherapy; and records of interventional procedures.
Adverse events: record adverse events from the time of signing the informed consent until the patient withdraws from the study. Survival status: If dead, try to record the cause of death.
Remarks: Blood biochemistry includes renal function, liver protein, bilirubin, liver enzymes and electrolytes.

5.4 Modalities of efficacy assessment

Radiological evaluation: Evaluation will be conducted according to the RECIST 1.1 criteria, including CT, MR, and bone scans. If a whole-body tumor PET-CT scan was performed before local treatment, it is acceptable. Follow-up visits were made every 12 weeks ± 7 days for 3 times after local therapy. If two or more local therapy regimens were received, the frequency of follow-up followed each therapy regimen individually.

Follow-up collection of information: vital signs, physical examination and adverse events, blood and imaging tests performed during the follow-up period are required to be collected, with no time window requirement. For the de-escalation Targeted therapy group, peripheral blood is collected for the analysis of ctDNA during follow-up.

Follow-up visits were performed during the ~~maintenance treatment /observation~~/de-escalation period, and imaging evaluations and hematology tests were performed according to test schedule.

Notification on patients to return to the hospital for follow-up in accordance with the timeframe specified in the protocol, and if for any reason the patient is unable to return, telephone call or other methods of communication is necessary to record the patient's detailed medical history, adverse events, test results, survival information, and other trial-requested content.

5.5 Patient efficacy assessment

Efficacy assessments included PFS, OS, safety, and quality of life scores. Primary efficacy indicators:

5.5.1 Primary endpoint

[redacted] In the de-escalation subgroup, PFS was defined from the TKI discontinuation to disease progression or death. Disease progression was defined according to RECIST 1.1 criteria. The date on that patients receive their last imaging evaluation while alive and progress-free is used as the cutoff.

5.5.2 Secondary endpoints

[redacted] OS: from the TKI discontinuation to time of death, or the last follow-up; TTNT: TTNT (time to next treatment) from the start of first-line (second-line) treatment to the start of next treatment. TTNT from the start of local therapy to the start of the next therapy.

5.5.3 Security assessment

The patient's physical examination, vital signs, adverse events, and laboratory test abnormalities are summarized. All adverse events were documented in accordance with NCI Common Terminology Criteria for Adverse Events (CTCAE) 4.0.

5.5.4 Quality of life scores

Assessing patients' quality of life scores (LCSS) and analyzing patients' quality of life.

6 Safety monitoring and reporting

6.1 Definition of Serious Adverse Event (SAE)

A serious adverse event is defined as an adverse event that occurs within any study phase (i.e., from the time the patient signs the study-related consent form, during the treatment period, and during the follow-up period) and meets one or more of the following criteria:

- lead to death
- Immediately life-threatening.
- Need for hospitalization or prolongation of current hospitalization
- Resulting in a persistent or significant disability/incapacity or significant impairment of the ability to perform normal life functions.
- Congenital anomalies or birth defects
- is a serious medical event that may jeopardize the patient's health or may require medical intervention to avoid either of these outcomes.

6.2 Reporting of Serious Adverse Events (SAEs)

All serious adverse events should be reported whether or not they are thought to be causally related to the study treatment or study procedure. All SAEs should be documented in the eCRF.

If a patient develops a serious adverse event during the course of the study, the researchers or other staff member of the research center should notify the regulatory body or the ethics committee of the research center immediately no later than 24 hours after report of the adverse event. The SAE must be fully described and documented on the case report form. All signs and symptoms of an adverse event must be reported in detail on the subject's case report form, including the nature of the sign or symptom, timepoint relative to the time of administration, duration, severity, possible association with therapeutic measures, necessary treatment medications, and outcome. All adverse events should be followed up until the adverse event resolves, or as determined by the principal investigator.

Any previously unreported events (in terms of nature, severity, or frequency) that are not present in the current investigator's brochure should be reported as unexpected adverse events.

6.3 No SAE Conditions

Hospital admissions due to disease progression

Hospitalization for planned local therapy.

related to medical insurance reasons.

7 Data collection and management

7.1 Traceability of data

The completion and transfer of Case Report Form (CRFs) are based on the most original records for research medical records, ensuring proper preservation. The case report form is taken from the study medical record and is completed by the investigators, who must complete the case report form for each enrolled case. The completed case report form is reviewed by the clinical supervisor and the first link is submitted to the data statistics unit for data entry and management. The content of the case report form will not be modified after the transfer of the first link.

7.2 Database design and creation

The design of the test database should be carried out in accordance with the data management plan, requiring functions such as double entry and checking, data trace tracking, and database locking. Pre-entry testing and data security and privacy testing are required before formal data entry.

Data entry and modification data entry and management is the responsibility of the statistics department of Guangdong Provincial People's Hospital. Specialized personnel will be appointed to manage the data and develop a data management plan. To ensure the accuracy of numerical data, double entries are made and proofread.

The data manager will complete the Data Rating Questionnaire (DRQ) and send a query to the investigator through the Clinical Supervisor, the investigator is warrant to response the query as soon as possible, and the data manager will correct the data based on the investigator's response, and may resend the DRQ again if necessary.

7.3 Data verification

The data manager performs data verification of the data and issues a data verification report, which is reviewed by the principal investigator, the sponsor, the statistical analyst and the data manager, and the data set for statistical analysis is determined before the data is locked.

7.4 Data quality control

Ensure that research data is true, accurate and reliable. Strictly control the whole process of data collection, processing, to statistical analysis and reporting. In accordance with the ALCOA+ data quality evaluation principles, ensure data traceability, completeness, consistency, and accuracy.

Prior to data collection, assess the establishment of collection fields, confirm that key fields have been collected, develop the appropriate CRF and EDC database architecture; establish standardization of data collection and entry, and ensure

consistency between the entered data and the data source. The entry of therapeutic drugs in the study will be coded at the time of entry into the database according to the WHO Drug Reference List, which is based on the Anatomical Therapeutic Chemical (ATC) classification. The patient's medical history/current illness and adverse events were also coded using terms from the International Medical Dictionary for Regulatory Activities (MedDRA).

Develop a comprehensive data quality management plan and establish key fields; develop a systematic quality control and manual quality control plan to ensure the completeness and accuracy of the information in the data sources and to reduce deficiencies and biases in the data sources themselves. EDC data collection must be completed for each patient, and the researcher is responsible for the final collection and reporting of all clinical, safety, and laboratory data entered on the EDC and any other data collection forms (source documents) for each patient, and for ensuring the accuracy, authenticity/originality, attribution, completeness, consistency, readability timeliness (contemporaneous), durability, and availability (when necessary) of these data. The researcher or authorized person must sign the paper CRF or source document to attest to the authenticity of the data collected, and any entry revisions made in the CRF or source document must be accompanied by the date, initials, and reason (if necessary), and should not obscure the original entry. Every data modification, update, review, audit, signature, etc. in the EDC system leaves a trace and can be viewed by authorized people. Control of bias and confounding variables

7.5 Control of bias and confounding variables

The choice of treatment for the patients in this study depended entirely on their condition and their own personal preferences. It was a non-randomized, open, non-placebo-controlled trial, closely mirroring a real-world medical environment.

7.5.1 Control methods for bias and confounding variables

Strictly control the inclusion or exclusion criteria of the study subjects, try to increase the response rate, reduce loss to follow-up, and assess patients who are lost to follow-up. Detailed records of the MDT's decision-making process and rationale for local treatment selection should be maintained, along with feedback on treatment outcomes.

Stratified analysis (by number of metastases), multifactorial analysis (cox regression study design), and propensity scores were used during statistical analysis to control for these biases.

The follow-up process followed the principles of observational research, i.e., the study was conducted under routine clinical practice, minimizing interventions in routine treatment and reflecting what happens in real clinical practice.

7.5.2 Control methods for information bias

Control and eliminate factors affecting the accuracy of information from research design and analysis, database management, etc. There are the control methods needed for information bias:

Study design phase: a rigorous, objective definition of a local therapy approach. Uniform and clear criteria for inclusion in the group. Purpose, significance and requirements of the study were clearly explained to the study participants to obtain their cooperation and support. researchers are rigorously trained to properly understand the significance, methodology and content of the survey, and are able to engage in data collection in a rigorous and objective manner. The researcher regularly checks the quality of the information and sets up quality control procedures.

Data collection phase: Methods of efficacy evaluation were harmonized as much as possible during the study phase. Collect patient information as well as questionnaires as objectively as possible.

8 Ethical Considerations and Management Procedures

8.1 Regulatory and Ethical Compliance

The clinical study design, implementation and reporting shall be carried out in accordance with the three-party coordinated guidelines of The International Council for Harmonization (ICH) of Technical Requirements for Pharmaceuticals for Human Use, relevant applicable local regulations (including EU Directive 2001/20/EC and 21 CFR) and ethical principles formulated in accordance with the "Declaration of Helsinki".

8.2 Responsibilities of the Investigator and IRB/IEC/REB

Before the start of the study, the study protocol and the proposed ICF must be reviewed and approved by the Institutional Review Board (IRB)/Independent Ethics Committee (IEC)/Research Ethics Board (REB) composed of relevant qualified personnel. Before the start of the study, the investigator is required to sign the signature page of the protocol to confirm that he/she agrees to carry out the study in accordance with these documents and all the instructions and procedures required by the study protocol, and to provide the relevant data and records to IRB/IEC/REB.

8.3 Informed Consent Procedures

Patients may be enrolled into the study only after eligible patients have provided written (in accordance with the law or regulations with witnesses present) ICF approved by IRB/IEC/REB.

Any study-specified procedures (i.e., all procedures specified in the study protocol) can only be performed after informed consent is obtained. The process of

obtaining the informed consent of the patient should be recorded in the patient's source document. The date on which the subject ICF is actually obtained will be recorded in the patient's CRF.

This study will use a separate document to provide the investigator with a proposed ICF, which will be implemented in accordance with the guidelines and regulations in the international unified standards for clinical trial management, and the ICF is considered to be applicable to this study. If the investigator proposes a revision to the ICF, the IRB/IEC/REB's approval must be obtained before using the revised ICF, and a copy of the approved version must be submitted to the sponsor and the monitor of each company.

8.4 Publication of Study Protocol and Results

The sponsor promises to report the study results in accordance with high ethical standards of publication, including timely publishing clinical trial results, regardless of the outcome. The sponsor shall ensure that the key design content of this study protocol is announced in a database open to the public, such as www.clinicaltrials.gov, before the start of the study.

8.5 Articles Published by the Investigator

Whether the results are favorable to the study drug, each company shall not veto the publication of any information collected or generated by investigator. However, in order to prevent inadvertent disclosure of confidential information and protect intellectual property, the investigator must notify the main responsible persons of each company before submission for publication or disclosure in other forms, so that each company has the opportunity to review and discuss the articles prepared for publication or other materials prepared for disclosure.

The sponsor supports the overall publication of the multicenter trial and does not support the separate publication of single-center data. The authorship will be decided by both parties or by tripartite agreement.

8.6 Storage, Records and Retention of Documents

According to ICH E6 GCP Section 4.9, relevant regulations and institutional requirements for protecting the confidentiality of subjects, each participating study site will keep appropriate medical history and study records related to this study. As part of the participation in this Trump clinical trial, each study site will allow the authorized representatives of the sponsor and regulatory authorities to inspect (and make copies if required by relevant laws) the clinical records to ensure high-quality study safety and to review, audit and evaluate.

Raw data includes all information, original records of clinical study results, observations or other clinical study activities necessary for reconstruction and

evaluation of the study. Examples of these source documents and data records include, but are not limited to, hospital records, clinical cases and office charts, laboratory records, data from automated instrument records, photocopies or copies of certifications to prove their accuracy and completeness, X-ray images, and clinical study-related subject documents and records stored at the pharmacy, laboratory and medical technology departments.

The clinical study staff will collect data at the study site and the principal investigator will perform on-site supervision. CRF is the main study data collection tool. The investigator should ensure the accuracy, integrity, timeliness and legibility of data recorded in the CRF and all reports required by the study. Data from the source documents and recorded in the CRF should be kept consistent with the data in the source documents. If there is a difference, a reasonable explanation should be provided. All data required by CRF must be recorded. All missing data must be reasonably explained. Any revision or amendment to the paper version of the CRF should be indicated with the date, the initials of the person making the revision and the reason (if necessary), and the original entered data should not covered. For eCRF, the system will set up a review trail. The investigator should keep a paper version of the CRF revision and amendment record.

The investigator/institution should retain the necessary documents (as required by ICH E6 Section 8) for conducting the clinical study and the study documents specified in the relevant applicable regulations and/or guidelines. The investigator/institution should take measures to prevent accidental or early destruction of these documents.

Starting from the date of completion of the clinical trial, necessary documents (written or electronic) shall be retained for at least fifteen (15) years, unless the study sponsor provides written materials to permit the disposal of these documents, or the retention time of these documents shall be extended in accordance with the requirements of current laws, regulations and/or guidelines.

8.7 Confidentiality of Study Documents and Patient Records

The study staff must ensure that the patient participates in the study anonymously, and the patient must not be identified by name in the documents submitted to each company. The signed ICF and patient enrollment records must be strictly confidential and can only be identified for patients at each study site.

9 Protocol Amendment

Any protocol amendment should be handed over to the sponsor for amendment, and a written protocol amendment shall be generated and submitted to the IRB/IEC/REB for approval.The revised protocol can only be implemented prior to IRB/IEC/REB approval if it is for ensuring the patient's safety. In this case, the investigator should inform the sponsor of the measures taken and report it to the

IRB/IEC/REB of the study site.

References

1. Siegel, R.L., K.D. Miller, and A. Jemal, Cancer statistics, 2016. CA Cancer J Clin, 2016. 66(1): p. 7-30.
2. Hellman, S. and R.R. Weichselbaum, Oligometastases. J Clin Oncol, 1995. 13(1): p. 8-10.
3. Suzuki, H. and I. Yoshino, Approach for oligometastasis in non-small cell lung cancer. Gen Thorac Cardiovasc Surg, 2016. 64(4): p. 192-6.
4. Shimada, Y., et al., Survival outcomes for oligometastasis in resected non-small cell lung cancer. Asian Cardiovasc Thorac Ann, 2015. 23(8): p. 937-44.
5. Pan, H., et al., A survey of stereotactic body radiotherapy use in the United States. Cancer, 2011. 117(19): p. 4566-72.
6. Rusthoven, K.E., et al., Multi-institutional phase I/II trial of stereotactic body radiation therapy for liver metastases. J Clin Oncol, 2009. 27(10): p. 1572-8.
7. Rusthoven, K.E., et al., Multi-institutional phase I/II trial of stereotactic body radiation therapy for lung metastases. J Clin Oncol, 2009. 27(10): p. 1579-84.
8. Wronski, M., et al., survival after surgical treatment of brain metastases from lung cancer: a follow-up study of 231 patients treated between 1976 and 1991. J Neurosurg, 1995. 83(4): p. 605-16.
9. Nakagawa, H., et al., Surgical treatment of brain metastases of lung cancer: retrospective analysis of 89 cases. J Neurol Neurosurg Psychiatry, 1994. 57(8): p. 950-6.
10. Kozower, B.D., et al., Special treatment issues in non-small cell lung cancer: Diagnosis and management of lung cancer, 3rd ed: American College of Chest Physicians evidence-based clinical practice guidelines. Chest, 2013. 143(5 Suppl): p. e369S-99S.
11. Patel, A.N., C.B. Simone, 2nd, and S.K. Jabbour, Risk factors and management of oligometastatic non-small cell lung cancer. Ther Adv Respir Dis, 2016. 10(4): p. 338-48.
12. Barone, M., et al., Oligometastatic non-small cell lung cancer (NSCLC): adrenal metastases. Experience in a single institution. Updates Surg, 2015. 67(4): p. 383-7.
13. Raz, D.J., et al., Outcomes of patients with isolated adrenal metastasis from non-small cell lung carcinoma. Ann Thorac Surg, 2011. 92(5): p. 1788-92; discussion 1793.
14. DeLuzio, M.R., et al., resection of oligometastatic lung cancer to the pancreas may yield a survival benefit in select patients--a systematic review. Pancreatology, 2015. 15(5): p. 456-62.
15. Lencioni, R., et al., Response to radiofrequency ablation of pulmonary tumours: a prospective, intention-to-treat, multicentre clinical trial (the RAPTURE study). Lancet Oncol, 2008. 9(7): p. 621-8.
16. Gomez, D.R., et al., Local consolidative therapy versus maintenance therapy or observation for patients with oligometastatic non-small-cell lung cancer without progression after first-line systemic therapy: a multicentre, randomised, controlled, phase 2 study. Lancet Oncol, 2016. 17(12): p. 1672-1682.
17. Gomez, D.R., et al., Local Consolidative Therapy Vs. Maintenance Therapy or Observation for Patients With Oligometastatic Non-Small-Cell Lung Cancer: Long-Term Results of a Multi-Institutional, Phase II, Randomized Study. J Clin Oncol, 2019. 37(18): p. 1558-1565.
18. Gundem, G., et al., The evolutionary history of lethal metastatic prostate cancer. Nature, 2015. 520(7547): p. 353-357.
19. Twyman-Saint Victor, C., et al., Radiation and dual checkpoint blockade activate non-redundant immune mechanisms in cancer. Nature, 2015. 520(7547): p. 373-7.
20. Chaudhuri AA, Chabon JJ, Lovejoy AF, et al. Early Detection of Molecular Residual

Disease in Localized Lung Cancer by Circulating Tumor DNA Profiling. Cancer Discov. 2017;7(12):1394-1403.

21. Moding EJ, Liu Y, Nabet BY, et al. Circulating Tumor DNA Dynamics Predict Benefit from Consolidation Immunotherapy in Locally Advanced Non-Small Cell Lung Cancer. Nat Cancer. 2020;1(2):176-183.

22. Zhang JT, Liu SY, Gao W, et al. Longitudinal Undetectable Molecular Residual Disease Defines Potentially Cured Population in Localized Non-Small Cell Lung Cancer. Cancer Discov. 2022;12(7):1690-1701.

23. Jung HA, Ku BM, Kim YJ, et al. Longitudinal Monitoring of Circulating Tumor DNA From Plasma in Patients With Curative Resected Stage I-IIIA EGFR Mutant-Non-Small Cell Lung Cancer. J Thorac Oncol. 2023.

24. Ding PN, Becker TM, Bray VJ, et al. The predictive and prognostic significance of liquid biopsy in advanced epidermal growth factor receptor-mutated non-small cell lung cancer: A prospective study. Lung Cancer. 2019;134:187-193.

25. Wang Z, Cheng Y, An T, et al. Detection of EGFR mutations in plasma circulating tumour DNA as a selection criterion for first-line gefitinib treatment in patients with advanced lung adenocarcinoma (BENEFIT): a phase 2, single-arm, multicentre clinical trial. Lancet Respir Med. 2018;6(9):681-690.

26. Zhang Q, Luo J, Wu S, et al. Prognostic and Predictive Impact of Circulating Tumor DNA in Patients with Advanced Cancers Treated with Immune Checkpoint Blockade. Cancer Discov. 2020;10(12):1842-1853.

27. Page RD, Drusbosky LM, Dada H, et al. Clinical Outcomes for Plasma-Based Comprehensive Genomic Profiling Versus Standard-of-Care Tissue Testing in Advanced Non-Small Cell Lung Cancer. Clin Lung Cancer. 2022;23(1):72-81.

28. Barlesi, F., et al., Randomized phase III trial of maintenance bevacizumab with or without pemetrexed after first-line induction with bevacizumab, cisplatin, and pemetrexed in advanced nonsquamous non-small-cell lung cancer: AVAPERL (MO22089). J Clin Oncol, 2013. 31(24): p. 3004-11.
