# Supplementary material for: Circulating Tumor DNA-Guided De-Escalation Targeted Therapy for Advanced Non−Small Cell Lung Cancer: A Nonrandomized Controlled Trial
Source: JAMA Oncol. 2024 Jun 13;10(7):932–40. doi: 10.1001/jamaoncol.2024.1779 (PMC12312504; doi:10.1001/jamaoncol.2024.1779)
Supplement: Supplement 2. — Statistical Analysis Plan [file jamaoncol-e241779-s002.pdf]

Statistical Analysis Plan of the de-escalation Group of CTONG1602

Tittle of CTONG1602:	Local Consolidative Therapy in Patients with Advanced Stage Non-small Cell Lung Cancer that Does Not Progress after Front Line Systemic Therapy
Tittle of the De-escalation arm:	Circulating Tumor DNA-guided Adaptive De-escalation Targeted Therapy for Advanced Non-Small Cell Lung Cancer Patients after Front Line TKI and Local Consolidative Therapy
Version:	Version: 1.0
Statistical Analysis Provider:	Guangdong Provincial People’s Hospital
Sponsor:	Guangdong Association of Clinical Trials/Chinese Thoracic Oncology Group (GACT/CTONG)
Principal investigator:	Zhen Wang

Cutoff Date: Nov 30, 2022

Table of Contents

1. Description of CTONG 1602 Study Design....3
2. Study Design of De-escalation TKI Group....3
2.1 Study Design....3
2.2 Sample Size Calculation of the De-escalation TKI Group....3
2.3 Grouping Method of the De-escalation TKI Group....4
3. Efficacy Assessment of the De-escalation TKI Group....4
3.1 Primary Endpoint....4
3.2 Secondary Endpoints....4
4. Statistical Analysis Study Design of De-escalation TKI Group....4
4.1 Baseline and Demographic Characteristics....5
4.2 Primary Endpoint Analysis....5
4.3. Secondary Endpoint Analysis....5
4.4 Security Analysis....5

1. Description of CTONG 1602 Study Design

This study is a prospective real-world study designed to explore the efficacy and survival benefit, as well as the safety of local therapy in progression-free patients with advanced Non-Small-Cell Lung Cancer (Stage IIIB, IV, 8th edition TNM staging) who received first-line systemic therapy (chemotherapy, targeted therapy, or immunotherapy), and received local therapy (surgery, radiotherapy, or interventional therapy). Patients eligible for enrollment were discussed in MDT to determine local therapy options and signed informed consent to enter this study for local therapy.

The exploratory Study de-escalation TKI subgroup: For driver gene positive patients who underwent surgery for all the primary and/or metastatic lesions with no detectable disease on radiographic imaging after surgery. ctDNA analysis will be performed for the patients. If the ctDNA, CEA and imaging remain negative, the patients will be enrolled into the de-escalation targeted therapy group; systemic targeted therapy will be discontinued, and if any one of these indicators becomes positive, then targeted therapy will be initiate.

2. Study Design of De-escalation TKI Group

2.1 Study Design

This is a polit part of CTONG 1602 with a plan to screen patients after front line TKI and local consolidative therapy (LCT) using NGS ctDNA platform. These patients will be enrolled in de-escalation TKI treatment group while the ctDNA is undetectable after LCT.

The primary endpoint is progression free survival (PFS), which is defined from the TKI discontinuation to disease progression or death. The other efficacy endpoints, such as overall survival (OS), time to next treatment (TTNT), will also be evaluated, so as safety and tolerability.

2.2 Sample Size Calculation of the De-escalation TKI Group

For the subgroup of De-escalation Targeted Therapy patients, the sample size was calculated using PASS software, version 15.0 (NCSS Statistical Software). As various TKI agents were used as first-line therapy in clinical practice and the historical control of PFS was set at 12 months, our study is designed with the expectation that a median of 8 months of PFS improvement (range of PFS, 12–20 months) would be observed. We aim to achieve a better median PFS than that of third-generation TKI used as first-line treatment.5 Assuming an enrollment time of 12 months and follow-up of 24 months, the sample size needed is determined to be 54 patients via the 1-sample log-rank test with a 1-sided significance level of 0.025 and 85% power. Considering a dropout rate of 10%, a total of 60 patients will be required.

2.3 Grouping Method of the De-escalation TKI Group

In respect to the study design, we anticipate that patients would resume treatment predicated on RECIST PD and/or the presence of a positive molecular biomarker. Consequently, certain patients may persist in a treatment break until the cut-off date. Therefore, we classified these patients based on the catalysts for their initial retreatment. To evaluate the significance of ctDNA in guiding the retreatment, three distinct groups are projected based on the first retreatment triggers of the enrolled patients. Group A, patients who have no positive indicators and continued treatment break; Group B, patients who initiate retreatment after displaying positive ctDNA and/or elevated CEA levels before RECIST-based PD; and Group C, patients with confirmed RECIST-based PD with or without positive molecular indicators during the first treatment break.

3. Efficacy Assessment of the De-escalation TKI Group

Efficacy assessments included PFS, OS, TTNT, and safety. Primary efficacy indicators:

3.1 Primary Endpoint

In the de-escalation subgroup, PFS was defined from the TKI discontinuation to disease progression or death. Disease progression was defined according to RECIST 1.1 criteria. The date on that patients receive their last imaging evaluation while alive and progress-free is used as the cutoff.

3.2 Secondary Endpoints

OS: from the TKI discontinuation to time of death, or the last follow-up; TTNT: TTNT (time to next treatment) from the start of first-line (second-line) treatment to the start of next treatment. TTNT from the start of local therapy to the start of the next therapy.

3.3 Security Assessment

The patient's physical examination, vital signs, adverse events, and laboratory test abnormalities are summarized. All adverse events were documented in accordance with NCI Common Terminology Criteria for Adverse Events (CTCAE) 4.0.

4 Statistical Analysis

The database was transferred to the statistical analysts for statistical analysis in accordance with the plan requirements. The statistical unit prepared a statistical

analysis report to the principal investigator who are obliged to complete the study results.

4.1 Baseline and Demographic Characteristics

Baseline demographic characteristics, baseline clinical characteristics, vital signs, and trial terminations were descriptively analyzed for all enrolled patients. For continuous measures, mean, standard deviation, range and median were calculated; absolute values, frequencies and percentages were calculated.

4.2 Primary Endpoint Analysis

PFS: The Kaplan-Meier method was used to describe the 25%, 75%, and median event occurrence times, along with a 95% two-sided confidence interval, and to plot the Kaplan-Meier survival curve up to the time of data cutoff. The COX proportional risk regression model was used for each covariate to test the influence factors related to the prognosis of recurrence in terms of hazard ratios (HR) and their estimated 95% two-sided confidence intervals.

4.3 Secondary Endpoint Analysis

OS and TTNT: The Kaplan-Meier method was used to describe the 25%, 75%, and median event occurrence times, along with a 95% two-sided confidence interval, and to plot the Kaplan-Meier survival curve up to the time of data cutoff. The COX proportional risk regression model was used for each covariate to test the influence factors related to the prognosis of recurrence in terms of hazard ratios (HR) and their estimated 95% two-sided confidence intervals.

4.4 Security Analysis

Adverse events and the most severe grades were analyzed descriptively according to the criteria of the NCI CTC-AE 4th edition. All adverse events occurring up to 28 days after the last administration of the drug or local therapy were recorded on the case report form. These adverse events were reported in the case form and summarized in a frequency table. Differences in the overall incidence of adverse events among groups and the incidence of adverse events at different response levels were compared using test distribution at a significance level of 0.05. Adverse events were summarized according to the severity of the event and its relationship to the drug or treatment. Descriptive summaries of laboratory test values focus on abnormal values.
