# Supplementary material for: Circulating Tumor DNA-Guided De-Escalation Targeted Therapy for Advanced Non−Small Cell Lung Cancer: A Nonrandomized Controlled Trial
Source: JAMA Oncol. 2024 Jun 13;10(7):932–40. doi: 10.1001/jamaoncol.2024.1779 (PMC12312504; doi:10.1001/jamaoncol.2024.1779)
Supplement: Supplement 3. — eFigure. Schematic flow chart of the study [file jamaoncol-e241779-s003.pdf]

Supplemental Online Content

Dong S, Wang Z, Zhang J, et al. Circulating Tumor DNA-guided De-escalation Targeted Therapy for Lung Cancer. *JAMA Oncol.* Published online June 13, 2024. doi:10.1001/jamaoncol.2024.1779

eFigure. Schematic flow chart of the study

This supplemental material has been provided by the authors to give readers additional information about their work.

© 2024 Dong S et al. *JAMA Oncol.*

eFigure. Schematic flow chart of the study

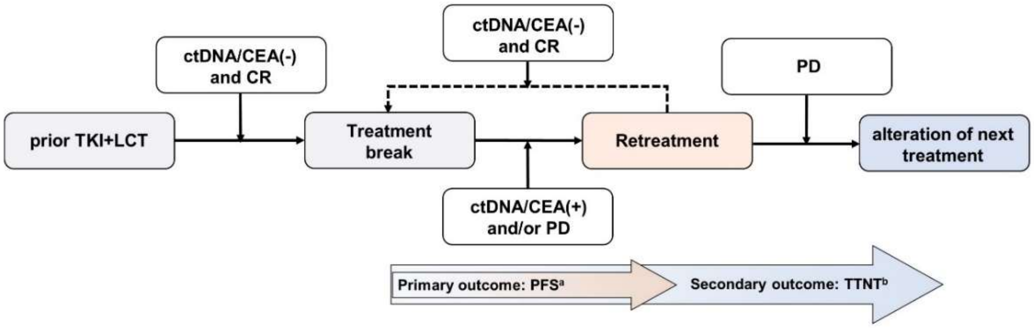

Abbreviations: TKI, tyrosine kinases inhibitor; LCT, local consolidative therapy; CEA, carcinoembryonic antigen; ctDNA, circulating tumor DNA; CR, complete response; PD, progressive disease; PFS, progression-free survival; TTNT, time to next treatment.

[a]PFS was defined as the time from treatment discontinuation to the initial identification of RECIST-criteria-based PD.

[b]TTNT was defined as the duration from the initiation of the first treatment break to the alteration of the next treatment.

© 2024 Dong S et al. *JAMA Oncol.*

Filename: COI240019supp3_edited.docx
Directory: C:\Users\sthollan\Desktop\SUPPLEMENTS
Template:
C:\Users\sthollan\AppData\Roaming\Microsoft\Templates\Normal.dot
m
Title:
Subject:
Author: song d
Keywords:
Comments:
Creation Date: 5/8/2024 11:49:00 AM
Change Number: 2
Last Saved On: 5/8/2024 11:49:00 AM
Last Saved By: SR Holland
Total Editing Time: 1 Minute
Last Printed On: 5/8/2024 11:49:00 AM
As of Last Complete Printing
Number of Pages: 2
Number of Words: 126 (approx.)
Number of Characters: 797 (approx.)
