# Supplementary material for: Circulating Tumor DNA-Guided De-Escalation Targeted Therapy for Advanced Non−Small Cell Lung Cancer: A Nonrandomized Controlled Trial
Source: JAMA Oncol. 2024 Jun 13;10(7):932–40. doi: 10.1001/jamaoncol.2024.1779 (PMC12312504; doi:10.1001/jamaoncol.2024.1779)
Supplement: Supplement 4. — eTable. Clinical characteristics and triggers of first retreatment for each patient [file jamaoncol-e241779-s004.pdf]

Dong S, Wang Z, Zhang J, et al. Circulating Tumor DNA-guided De-escalation Targeted Therapy for Lung Cancer. *JAMA Oncol.* Published online June 13, 2024. doi:10.1001/jamaoncol.2024.1779

eTable. Clinical characteristics and triggers of first retreatment for each patient

Patient ID	Group	Smoking	Ancestry	Race	Ethnicity	Gender	Age	Stage	Somatic driver gene mutations	Somatic passenger gene mutations	Elevated baseline CEA	Target Drug	Location of Metastasis	LCT	TKI time before treatment break (months)	Total treatment break duration (months)	Trigger of first retreatment		
P07	Group A	No	East Asian ancestry	Asian	Han Chinese	Female	60	IV	EGFR L858R	MDC1, PARK2, SNTG1,ATRX, AXIN1, BRCA1, ERCC3	No	EGFR-TKI I + EGFR-TKI III	Brain	Surgery & Radiotherapy	5.1	26.8	None	No	No
P33	Group A	No	East Asian ancestry	Asian	Han Chinese	Male	66	IV	ALK fusion	FAM123B,ARID1A, ATM, FLCN, LRP1B	No	ALK-TKI II	Bone	Surgery	17.3	16.0	None	No	No
P25	Group A	No	East Asian ancestry	Asian	Han Chinese	Male	21	IV	EGFR 19del	TP53	No	EGFR-TKI II	Bone	Surgery	6.3	22.6	None	No	No
P64	Group A	No	East Asian ancestry	Asian	Han Chinese	Male	40	IV	EGFR 19del	CYP17A1, FAT2, HDAC2, HIST1H1C, MERTK, MLL, MLL3, PIK3C2G, PRKCI, SI, TOP2A,ASXL1, CCNE1, CTNNB1, ERBB3, FAM135B, FANCA, KAT6A, MET, MPL, MTOR, PMS2, RAD21, SPEN, TERT, TP53, ZFHX3	No	EGFR-TKI I	Bone	Surgery	40.8	8.1	None	No	No
P72	Group B	No	East Asian ancestry	Asian	Han Chinese	Female	42	IV	EGFR 19del	None	No	EGFR-TKI III	Bone	Surgery	18.9	1.0	EGFR	No	No
P48	Group C	Yes	East Asian ancestry	Asian	Han Chinese	Male	52	IV	EGFR 19del	APC, BCL11B, BRD3, TP53	No	EGFR-TKI II	Bone,Lung	Surgery	14.4	4.3	None	No	Yes
P51	Group A	No	East Asian ancestry	Asian	Han Chinese	Male	68	IV	EGFR L858R	NOTCH4,ATR,TP53	Yes	EGFR-TKI I	Bone	Surgery	36.3	10.9	None	No	No
P68	Group B	No	East Asian ancestry	Asian	Han Chinese	Male	39	IV	EGFR L858R	DIS3, TMEM132D, LRP1B, TP53	No	EGFR-TKI II	Bone	Surgery	5.1	5.0	EGFR, TP53, TMEM132D	No	No
P61	Group A	Yes	East Asian ancestry	Asian	Han Chinese	Female	57	IV	EGFR L858R	BRCA1, CDKN2C,RBM10, TP53	No	EGFR-TKI II	Bone	Surgery	9.1	9.7	None	No	No
P69	Group B	No	East Asian ancestry	Asian	Han Chinese	Female	52	IV	EGFR 19del	None	No	EGFR-TKI III	Bone	Surgery	14.7	3.0	EGFR	No	No
P50	Group B	No	East Asian ancestry	Asian	Han Chinese	Female	58	IV	EGFR 19del	FANCD2, TP53	No	EGFR-TKI II	Bone, Lung	Surgery & Radiotherapy	11.7	8.5	TP53	No	No
P46	Group B	No	East Asian ancestry	Asian	Han Chinese	Male	42	IV	EGFR 19del	None	No	EGFR-TKI III	Bone, Lung, Brain	Surgery	21.4	2.2	EGFR	No	No
P12	Group C	No	East Asian ancestry	Asian	Han Chinese	Female	47	IV	EGFR L858R+T790M	APC	Yes	EGFR-TKI II + EGFR-TKI III	Bone, Lung, Brain	Surgery	15.7	2.9	EGFR	No	Yes
P53	Group B	No	East Asian ancestry	Asian	Han Chinese	Male	30	IV	EGFR 19del	IRF2, SI,CDC73, ERBB3, KRAS, MYD88	No	EGFR-TKI I + EGFR-TKI III	Brain	Surgery	11.2	1.5	EGFR	No	No
P01	Group C	Yes	East Asian ancestry	Asian	Han Chinese	Male	69	IV	EGFR L858R	None	No	EGFR-TKI I	Brain	Radiotherapy	2.1	12.0	None	No	Yes
P06	Group C	No	East Asian ancestry	Asian	Han Chinese	Female	37	IV	EGFR 19del+T790M	PARP1,TP53	No	EGFR-TKI III	Brain, Bone,Lung	Surgery	26.4	12.8	None	No	Yes

P47	Group A	No	East Asian ancestry	Asian	Han Chinese	Male	43	IV	EGFR L858R	FANCL, FANCM, FAT2, HOXB13, MLL, NFKBIA, RAD50, ROBO3, ABL1, AR, ATM, BRCA2, ETV1, KDR, NF1, NTHL1, TP53	No	EGFR-TKI I	Diaphragm	Surgery	4.0	12.4	None	No	No
P14	Group B	No	East Asian ancestry	Asian	Han Chinese	Female	56	IV	EGFR 19del	CARD11, CTNNB1, MED12, POT1, PTPRD, TP53	No	EGFR-TKI I	Lung	Surgery	8.6	13.7	NOTCH 4	No	No
P23	Group C	No	East Asian ancestry	Asian	Han Chinese	Male	26	IV	EGFR 19del	MLL3, SPTA1, RB1, TP53	No	EGFR-TKI I	Lung	Surgery	9.8	1.8	POT1	No	Yes
P40	Group B	No	East Asian ancestry	Asian	Han Chinese	Female	44	IV	EGFR 19del	FAM123B, POM121L12, MAP2K4	No	EGFR-TKI I + EGFR-TKI III	Lung	Surgery	6.2	10.0	EGFR	No	No
P10	Group B	Yes	East Asian ancestry	Asian	Han Chinese	Male	59	IV	EGFR 19del	ARID5B, FANCM, PIK3CG	No	EGFR-TKI II	Lung	Surgery	9.3	14.0	HDAC4	No	No
P09	Group C	No	East Asian ancestry	Asian	Han Chinese	Female	56	IV	EGFR 19del+T790M	COL5A3, CYP1A1, FANCM, RECQL, LRP1B, NF1, TP53	No	EGFR-TKI I + EGFR-TKI III	Lung	Surgery	6.3	11.8	None	No	Yes
P71	Group B	No	East Asian ancestry	Asian	Han Chinese	Female	55	IV	EGFR 19del+T790M	AURKA, CTNNA1, NAV3, EML4, KIT, MAP3K13, RUNX1, TP53	No	EGFR-TKI I + EGFR-TKI III	Lung	Surgery	15.2	3.3	EGFR	No	No
P65	Group A	No	East Asian ancestry	Asian	Han Chinese	Female	63	IV	EGFR L858R	GATA4,BRAF, CSMD3, MAP2K4, PIK3CA	No	EGFR-TKI III	Lung	Surgery	65.1	6.9	None	No	No
P66	Group B	No	East Asian ancestry	Asian	Han Chinese	Male	69	IV	EGFR L858R	GATA4, GNA13,BRCA2, CREBBP, CSMD3, CTNNB1,ELF3, PIK3CA, PTPRD, TP53	No	EGFR-TKI III	Lung	Surgery	8.8	2.3	EGFR	No	No
P17	Group A	Yes	East Asian ancestry	Asian	Han Chinese	Male	50	IV	EGFR L858R	None	No	EGFR-TKI I	Lung	Surgery	20.1	26.5	None	No	No
P39	Group B	No	East Asian ancestry	Asian	Han Chinese	Female	41	IV	EGFR 19del+T790M	DOT1L,ABL1, BCOR, PREX2, PSIP1, RB1, TCF7L2, TP53, TSC2	Yes	EGFR-TKI II + EGFR-TKI III	Lung	Surgery	19.6	7.5	EGFR, PTEN, APC	No	No
P04	Group B	Yes	East Asian ancestry	Asian	Han Chinese	Male	53	IV	EGFR 19del	MSH3, PCK1, BCOR, CDC73, GNA11, KDM5C, PIK3CB, PMS2, SMARCA4	No	EGFR-TKI I	Lung, Brain	Surgery	30.5	5.4	ARID1B	No	No
P28	Group A	No	East Asian ancestry	Asian	Han Chinese	Female	46	IVA	EGFR L858R+T790M	INPP4B, PIK3CA, RAC1	Yes	EGFR-TKI I + EGFR-TKI III	Lung, Ovary, Pleura	Surgery	44.4	28.1	None	No	No
P05	Group C	No	East Asian ancestry	Asian	Han Chinese	Female	69	IIIA	EGFR 19del	ARID1B, NPM1, TP53	No	EGFR-TKI I	Lymph	Surgery	11.2	16.2	None	No	Yes
P22	Group A	Yes	East Asian ancestry	Asian	Han Chinese	Male	58	IIIA	EGFR 19del	TP53	Yes	EGFR-TKI II	Lymph	Surgery	3.8	23.4	None	No	No
P20	Group A	Yes	East Asian ancestry	Asian	Han Chinese	Male	62	IIIA	EGFR L858R	None	No	EGFR-TKI I	Lymph	Radiotherap y	36.0	24.0	None	No	No
P34	Group C	No	East Asian ancestry	Asian	Han Chinese	Male	62	IIIB	ALK fusion	CASP8, TP53	No	ALK-TKI II	Lymph	Surgery	5.0	2.6	None	No	Yes
P31	Group B	No	East Asian ancestry	Asian	Han Chinese	Female	39	IIIB	EGFR 19del	PIK3CA, TP53	No	EGFR-TKI I	Lymph	Surgery & Radiotherap y	4.9	11.9	EGFR, PIK3CA	No	No
P16	Group B	No	East Asian ancestry	Asian	Han Chinese	Female	55	IIIB	EGFR L858R	SMAD4	No	EGFR-TKI I	Lymph	Surgery	3.9	5.4	EGFR	No	No

P60	Group C	No	East Asian ancestry	Asian	Han Chinese	Female	66	IIIB	EGFR L858R+T790M	STAT4, TIPARP,, SMARCA4, TP53	Yes	EGFR-TKI III	Lymph	Surgery	30.2	4.2	None	No	Yes
P43	Group A	No	East Asian ancestry	Asian	Han Chinese	Female	32	IV	ALK fusion	MAP2K4	Yes	ALK-TKI II	Pleura	Surgery	34.7	12.0	None	No	No
P18	Group C	No	East Asian ancestry	Asian	Han Chinese	Female	56	IV	EGFR 19del	EPHA5, FANCM, TRPC5,CARD11, CTCF,JAK3	No	EGFR-TKI III	Pleura	Surgery	3.0	17.5	EGFR	No	Yes
P19	Group C	No	East Asian ancestry	Asian	Han Chinese	Female	65	IV	EGFR 19del	EPHA5, HDAC1, HSD3B1, INPPL1, NOTCH3, PIK3CD, ASXL1, DNMT3A, FAT1, FOXP1, GATA3, GLI1, KMT2C, MLH1, TOP1, WWTR1	Yes	EGFR-TKI III	Pleura	Surgery	7.2	7.5	None	No	Yes
P44	Group A	No	East Asian ancestry	Asian	Han Chinese	Female	50	IV	EGFR 19del	CHN1, EPHA5, FZD1, BARD1, CTNNB1, KEAP1, MET, SMAD2, TLX3, TP53	Yes	EGFR-TKI I	Pleura	Surgery	13.3	20.7	None	No	No
P15	Group B	No	East Asian ancestry	Asian	Han Chinese	Male	54	IV	EGFR L858R	NCOR1	No	EGFR-TKI II	Pleura	Surgery	3.0	15.1	EGFR, TP53	No	No
P08	Group B	No	East Asian ancestry	Asian	Han Chinese	Female	51	IV	EGFR 19del	HIST1H2AG, MLH3,ARID1A, TP53	Yes	EGFR-TKI II	Pleura,lung	Surgery	12.3	10.2	EGFR	No	No
P02	Group B	Yes	East Asian ancestry	Asian	Han Chinese	Male	68	IV	EGFR L858R	MLL2, PDK1, RICTOR,CDK4, CTCF, KIF5B, RNF43, TP53	Yes	EGFR-TKI I	Pleura	Surgery	13.2	20.0	EGFR	No	No
P37	Group B	No	East Asian ancestry	Asian	Han Chinese	Male	48	IVA	EGFR 19del	SUFU	Yes	EGFR-TKI II	Pleura	Surgery	20.5	11.5	RET,PDK1L1, MLL	No	No
P63	Group B	No	East Asian ancestry	Asian	Han Chinese	Female	51	IV	EGFR 19del+T790M	C11orf30, RUNX2,APC, ATR, POLD1, SYK	No	EGFR-TKI I + EGFR-TKI III	Pleura, Lung	Surgery	4.2	8.5	APC, TP53, EGFR	No	No
P42	Group B	No	East Asian ancestry	Asian	Han Chinese	Male	65	IV	ROS1 fusion	ACIN1, MLL, MLL2, MSH3,CDH11, CDKN2C, JUN, MAP2K4,	No	ALK-TKI I	Pleura, Lung,Bone	Surgery	24.4	11.5	ROS1	No	No
P67	Group B	No	East Asian ancestry	Asian	Han Chinese	Female	59	IV	EGFR L858R	TP53	Yes	EGFR-TKI III	Pleura,Pericardium	Surgery	6.5	3.0	EGFR, TP53	No	No
P49	Group C	No	East Asian ancestry	Asian	Han Chinese	Female	52	IV	EGFR 19del	C11orf30, EPHB1, MLL3, NFKBIA,CHD4, JAK3, PIK3CA, PTEN, RB1, TP53	Yes	EGFR-TKI II	Bone	Surgery	16.2	6.7	EGFR	Yes	Yes
P03	Group C	No	East Asian ancestry	Asian	Han Chinese	Female	64	IV	EGFR 19del	ATM, CDK4, PIM1, TP53, ZFHX3	Yes	EGFR-TKI II	Lung	Surgery	13.3	9.8	None	Yes	Yes
P55	Group B	No	East Asian ancestry	Asian	Han Chinese	Female	65	IV	EGFR 19del	ACVR1B, C1S,CYLD, MAP2K1, MAP2K4, RBM10, ROS1, TP53	Yes	EGFR-TKI III	Lung	Surgery	11.7	5.3	None	Yes	No
P70	Group B	Yes	East Asian ancestry	Asian	Han Chinese	Male	64	IV	EGFR 19del+T790M	MLL4,EPHA7, TP53	Yes	EGFR-TKI III	Lung	Surgery	3.4	2.8	EGFR	Yes	No
P21	Group C	No	East Asian ancestry	Asian	Han Chinese	Male	52	IV	EGFR L858R	MPL, TP53	Yes	EGFR-TKI II + EGFR-TKI III	Lung	Surgery	9.5	3.0	EGFR	Yes	Yes
P30	Group C	Yes	East Asian ancestry	Asian	Han Chinese	Male	49	IV	EGFR 19del	EPCAM, EPHB6, GRIN2A, RB1, TET2, TP53	Yes	EGFR-TKI I	Lung, Bone, Brain	Surgery	9.6	3.9	EGFR	Yes	Yes

P36	Group B	No	East Asian ancestry	Asian	Han Chinese	Male	72	IIIA	EGFR L858R	TGFBR1	Yes	EGFR-TKI II	Lymph	Surgery&Radiotherapy	3.4	13.6	EGFR	Yes	No
P29	Group B	No	East Asian ancestry	Asian	Han Chinese	Female	62	IV	EGFR L858R	BRCA1, CTNNB1, TP53	Yes	EGFR-TKI II	Pericardium	Surgery	3.0	20.6	EGFR,FLT1	Yes	No
P13	Group B	No	East Asian ancestry	Asian	Han Chinese	Female	45	IV	EGFR 19del	None	Yes	EGFR-TKI I	Pleura	Surgery	30.6	12.1	None	Yes	No
P11	Group B	No	East Asian ancestry	Asian	Han Chinese	Female	75	IV	EGFR L858R	CTNNA1, FLNC, HDAC4, IRS1, TYR,APC, BCOR, LRP1B, MEN1, MET, MTOR, NF1, NTRK1, PTPRD, RBM10, SMAD3, STAG2	Yes	EGFR-TKI I	Pleura	Surgery	4.4	15.2	None	Yes	No
P24	Group B	No	East Asian ancestry	Asian	Han Chinese	Female	68	IV	EGFR 19del	BRD2, ATRX, BCOR, FANCE, KDM6A, PTEN, TP53	Yes	EGFR-TKI II	Pleura, Bone	Surgery	7.3	6.7	None	Yes	No
P26	Group B	No	East Asian ancestry	Asian	Han Chinese	Female	41	IV	EGFR 19del	CCNE1, LRP1B, NF1, STAT3, TP53	Yes	EGFR-TKI II	Pleura, Bone	Surgery	9.9	5.4	None	Yes	No
P54	Group B	No	East Asian ancestry	Asian	Han Chinese	Male	68	IVA	EGFR 19del	MLL2, RINT1, KDM5C, MAP3K1, SPOP	Yes	EGFR-TKI III	Pleura, Lung	Surgery	7.3	6.5	EGFR, NF1, KDM6, PTCH1	Yes	No
