# Supplementary material for: Circulating Tumor DNA-Guided De-Escalation Targeted Therapy for Advanced Non−Small Cell Lung Cancer: A Nonrandomized Controlled Trial
Source: JAMA Oncol. 2024 Jun 13;10(7):932–40. doi: 10.1001/jamaoncol.2024.1779 (PMC12312504; doi:10.1001/jamaoncol.2024.1779)
Supplement: Supplement 5. — Data Sharing Statement [file jamaoncol-e241779-s005.pdf]

Data Sharing Statement

Dong. Circulating Tumor DNA-Guided De-Escalation Targeted Therapy for Advanced Non–Small Cell Lung Cancer. *JAMA Oncol*. Published June 13, 2024.
doi:10.1001/jamaoncol.2024.1779

Data
Data available: Yes
Data types: Deidentified participant data, Participant data with identifiers
How to access data: dsong@aliyun.com
When available: With publication

Supporting Documents
Document types: Statistical/analytic code
How to access documents: dsong@aliyun.com
When available: With publication

Additional Information
Who can access the data: researchers
Types of analyses: for any purpose
Mechanisms of data availability: after approval of a proposal
